# Cross-Platform External Validation of a Locked 12-Gene Prognostic Panel in Lung Adenocarcinoma Benchmarked Against Pathological Stage

**DOI:** 10.3390/genes17101264

**Published:** 2026-10-11

**Authors:** Faris Alrumaihi

**Affiliations:** Department of Medical Laboratories, College of Applied Medical Sciences, Qassim University, Buraydah 51452, Saudi Arabia; f_alrumaihi@qu.edu.sa

**Keywords:** lung adenocarcinoma, cross-platform transportability, fixed gene panel, external validation, survival analysis, calibration, model comparison, pathological stage, risk stratification, incremental value

## Abstract

Background/Objectives: A prognostic gene panel is clinically useful only if its risk ranking survives transfer across measurement platforms and adds information to pathological stage. We evaluated the cross-platform transportability of a 12-gene panel selected in TCGA-LUAD and fixed before external scoring, separating panel performance from model choice and benchmarking it against stage. Methods: Selection used 504 TCGA patients and genes measurable on all validation platforms. Five molecular survival learners received identical gene inputs; age-and-sex and stage-based Cox models served as clinical comparators. External evaluation included 1066 patients in GSE72094, GSE68465 and GSE31210. Discrimination, paired model differences, prognostic value beyond stage, calibration and selection stability were assessed. Results: SurvivalPFN achieved Harrell C-indices of 0.631 (95% CI 0.572–0.689), 0.583 (0.541–0.624) and 0.665 (0.581–0.750), respectively, and regularized Cox regression on the same genes performed comparably (paired differences 0.005–0.011). The panel score was associated with survival independently of stage, with hazard ratios per standard deviation of 1.36 (95% CI 1.12–1.65) in GSE72094 and 1.56 (1.11–2.17) in GSE31210. Stage alone, however, discriminated at least as well as the panel alone in every cohort. Adding the panel to stage gave the highest C-indices observed in these cohorts, 0.663 and 0.718, against 0.634 and 0.678 for stage alone, but neither gain was statistically significant. Panel-defined risk groups separated overall survival in all three cohorts (hazard ratios 2.39, 1.43 and 3.65). Predicted 3-year risk matched observed risk in both primary cohorts (observed-to-predicted ratios 1.02 and 1.00), whereas TCGA-derived conformal correction improved calibration only in GSE68465. Results were consistent across learners and in sensitivity analyses of cohort overlap, histology and gene expression level. Conclusions: The fixed panel carried prognostic information that transported across RNA-sequencing and microarray platforms and complemented pathological stage, but it did not outperform stage. Its prospective evaluation should therefore be as an adjunct to stage, with local recalibration.

## 1. Introduction

Lung cancer is the leading cause of cancer death worldwide, and adenocarcinoma is its most common histological subtype [1,2]. Outcomes vary widely among patients with similar clinicopathological features, and The Cancer Genome Atlas showed that lung adenocarcinoma (LUAD) comprises heterogeneous somatic, copy-number and transcriptional programs rather than a single molecular state [3]. Stage remains the principal determinant of management [4], and the introduction of adjuvant osimertinib and perioperative chemoimmunotherapy has changed the context in which postoperative risk is estimated [5,6]. Reporting standards for tumor marker studies were set out in REMARK [7], and the archived-specimen framework established that retrospective data can support clinical claims when the analysis is specified in advance [8]. The 14-gene assay for non-squamous non-small-cell lung cancer is among the few expression panels to have reached international validation [9], and the Director’s Challenge evaluated expression-based survival prediction blind and across sites, establishing that clinical covariates set the benchmark any signature has to beat [10]. Public cohorts, including GSE72094, GSE68465 and GSE31210, are widely reused for signature discovery [11,12,13,14], and panels built from tumor-microenvironment genes, ferroptosis regulators, RNA-binding proteins and immune programs are reported frequently [15,16,17,18,19]. Such studies typically screen a high-dimensional discovery set, fit a short gene list and report discrimination in one or two retrospective cohorts. Few such signatures have been shown to add information to stage and other standard clinicopathological predictors, and a systematic review concluded that the published lung cancer signatures it examined were not ready for clinical use [20]. Random gene sets can also be associated with outcome [21], so a new panel is best judged against stage and against a random-signature reference. Two properties of the data determine whether such estimates generalize. Platform and probe differences shift measured expression values [22], and gene lists drawn from correlated features are unstable, so small perturbations of the discovery sample can yield a different list with similar predictive performance [23]. Three further concepts are distinct but often conflated. Discrimination is not calibration: a score can rank patients correctly while assigning inaccurate absolute risk, which is what threshold-based decisions depend on [24]. External validation is not a random split of the discovery set; it requires a change in laboratory and population without reintroducing external outcomes into feature selection [25,26]. Differential expression between tumor and normal tissue indicates biological relevance rather than clinical validity.

Machine-learning methods carry their own evaluation requirements. DeepSurv implements the Cox partial likelihood within a neural network [27], and tree ensembles capture interactions that linear models do not [28]. Prior-data fitted networks learn an inference procedure across many synthetic tasks rather than fitting weights to each new dataset, and perform well on small tabular problems [29]. SurvivalPFN adapts this approach to right-censored outcomes and performs in-context survival inference [30]. It is currently available as a preprint that has not undergone peer review. Pretrained in-context learners of this kind are often called tabular foundation models; we use that term only in this technical sense, without implying clinical generality. Whether amortized inference benefits transcriptomic oncology, where cohorts are small relative to the number of features, can only be settled by comparing it with a well-regularized Cox model on identical inputs, in cohorts withheld from every development decision. Conformalized survival distributions adjust individual survival curves while accounting for censoring [31], and how they behave when transported from RNA sequencing to microarrays is the corresponding open question for calibration.

The primary question had three parts: whether a panel selected in TCGA retains prognostic performance when transferred to independent populations measured on different platforms; whether that performance adds to pathological stage, the benchmark any prognostic panel for resected LUAD has to exceed; and how far it depends on the learner. We evaluated the same 12 genes across five molecular learners, with an age and sex Cox comparator, and assessed discrimination separately from survival-distribution calibration. The gene list, model specifications and cohort roles were recorded before external scoring. Stage-based comparators, a random-signature reference and the calibration of the conventional Cox model were specified after the lock and are reported as such. We report all six prespecified interpretation criteria, including those not met, and separate the five substantive criteria from the one procedural step.

## 2. Materials and Methods

### 2.1. Study Design, Intended Use and Cohort Roles

This retrospective computational study used public, de-identified molecular and clinical data. TCGA-LUAD was the sole discovery cohort. GSE72094 and GSE68465 were designated primary external validation cohorts. GSE31210 was designated a stage I–II stress-test cohort because of its low event count and restricted stage distribution [12,13]. All cohort roles were fixed before external scoring. The intended use was research-stage estimation of overall-survival risk after LUAD diagnosis or resection, specifically as a candidate adjunct to pathological stage for ranking patients after resection. The value of the panel was therefore judged by the prognostic information it adds to stage. The study was not designed to select systemic therapy, predict treatment response, replace TNM staging or set surveillance intervals. External outcomes were not used to choose the gene universe, select the panel, tune hyperparameters, alter preprocessing or decide which locked model to report. XGBoost Cox [32], SurvivalPFN augmented with age and sex, and stage-only Cox models were specified after the primary lock and are reported as sensitivity analyses. Additional post-lock analyses, including the stage benchmark, the random-panel reference and the calibration of panel Cox, are described in Section 2.15.

### 2.2. Ethics Statement

All data analyzed in this study are publicly available, de-identified secondary data obtained from the NCI Genomic Data Commons and NCBI Gene Expression Omnibus. No new human participants, human samples or animal experiments were involved. Ethical review and approval were waived for this study because it used only public, de-identified secondary data, and patient consent was waived on the same basis; informed consent had been obtained from participants in the original studies that generated these datasets. All analyses were performed in accordance with relevant guidelines and regulations, including the Declaration of Helsinki and the data-use policies of the NCI Genomic Data Commons and NCBI Gene Expression Omnibus.

### 2.3. Input Audit and Raw-Data Preservation

The TCGA input files were audited directly. The STAR-count matrix contained 60,660 gene rows and 601 aliquot columns; the accompanying variance-stabilized matrix covered the same gene universe and aliquots; the metadata contained 601 rows and 92 fields [33]. Source files were left unchanged, derived objects were written to separate directories, and SHA-256 checksums were recorded for each. TCGA barcodes were parsed into patient, sample, vial and aliquot components. The metadata described 540 primary tumors, 59 solid-tissue normals and two recurrent tumors from 518 patients. Twelve patients contributed more than one primary-tumor aliquot. To prevent pseudoreplication, one primary tumor per patient was selected deterministically: the preferred vial first, then the largest raw library size where a tie remained. Normal samples were excluded from survival modeling and used only in the post-selection paired analysis.

### 2.4. Endpoint and Eligibility

Overall survival was the only modeled endpoint. For deceased patients, survival time was days to death; otherwise, it was days to last follow-up, with an event indicator of one denoting death. Patients were excluded when no primary tumor was available, when follow-up was missing or when time was not positive. Age and sex were retained as a transportable clinical comparator, and pathological stage was retained where recorded. Stage and survival were then cross-checked against the curated TCGA Pan-Cancer Clinical Data Resource (TCGA-CDR) [34]. The CDR supplied pathological stage for 496 of the 504 patients, compared with 384 in the flattened GDC field, and CDR stage was used in every stage analysis. Its survival status agreed with the GDC-derived values for all 504 patients and its survival time for 503. Overall survival in TCGA is measured from initial pathological diagnosis. The GEO records do not state the time origin uniformly, but all three external cohorts consist of resected tumors, so their follow-up begins at or shortly after surgery. This difference cannot be removed with the available data. The flattened local diagnosis field contained non-LUAD labels despite a consistent TCGA-LUAD project identifier and consistent barcode provenance. Because the project source and barcodes agreed and only the derivative field did not, diagnosis was retained as an audit variable and was not used for inclusion or exclusion. TCGA-CDR classified all 504 patients as lung adenocarcinoma. Eligibility and aliquot decisions were written to a machine-readable audit table.

### 2.5. Annotation, Count Filtering and Outcome-Blind Transformation

STAR count rows were mapped to GENCODE release 36 on GRCh38.p13 by exact Ensembl identifier [33,35]. Version suffixes and pseudoautosomal _PAR_Y identifiers were handled explicitly. Genes were retained when they carried at least 10 counts in at least 51 of the eligible patients. DESeq2 variance-stabilizing transformation used design ~1 with blind = TRUE, so that survival outcome, stage and external-cohort information could not influence normalization [36].

The transformed matrix was restricted to protein-coding genes. Where several Ensembl records mapped to one symbol, the higher-variance feature was retained. The 2000 genes with the greatest variance in TCGA were selected without reference to outcomes.

### 2.6. External Cohorts and Cross-Platform Harmonization

GEO series matrices, phenotype annotations and platform records were downloaded from the accession pages [14]. GSE72094 is a resected LUAD cohort with molecular annotation reported by Schabath et al. [11]. GSE68465 originates from the Director’s Challenge multi-site blinded validation study [10]. GSE31210 contains predominantly early-stage Japanese LUAD [12,13]. Survival eligibility was reconstructed from each cohort’s documented fields. Probe identifiers were mapped to gene symbols using the corresponding platform annotation, and control samples were excluded where applicable. Probes annotated to more than one gene symbol were discarded, and the remaining probes for each symbol were collapsed to their per-sample median. A cohort was log2(x + 1) transformed when the 99th percentile of its downloaded values exceeded 100, which indicates a linear intensity scale. This applied to the MAS5-processed GSE68465 and GSE31210 data, whereas GSE72094 was supplied on a log scale. Cohorts were not pooled, and no outcome-aware batch correction was applied [22]. Each cohort was standardized independently, gene by gene, using its own median and median absolute deviation (scaled by 1.4826). The standard deviation was substituted when the median absolute deviation was zero, and a unit scale when both were zero. Centering each cohort on its own median removes between-cohort differences in average expression and, with them, differences in average predicted risk. This preserves within-cohort ranking but limits the transport of absolute risk, which was examined in the calibration analyses. The discovery universe was defined as the genes measurable in TCGA and on all three external platforms. External expression values constrained technical measurability, but no external outcome was accessed during feature selection. This design evaluates transport across the combined differences in assay platform, population, case mix and treatment era; it does not isolate a causal platform effect. GSE72094 and the 79 GSE68465 patients contributed by the H. Lee Moffitt Cancer Center (site code HLM) come from the same institution, so possible patient overlap was examined (Section 2.15).

### 2.7. Survival Screening, Elastic-Net Selection and Stability Analysis

Within TCGA, the shared genes were ranked by univariable survival association, and the leading 250 entered an elastic-net Cox proportional-hazards model [37,38]. The mixing parameter was fixed at 0.9. The regularization path was evaluated by cross-validation and the penalty chosen by the one-standard-error rule, giving a penalty of 0.0597023. Selection stability was assessed with 200 patient-level bootstrap replicates [23]. In each replicate, patients were drawn with replacement and the elastic-net fit was repeated on the 250 screened genes at the fixed penalty. The univariable screen itself was performed once in the full discovery cohort and was not repeated within replicates. Because this can make selection frequencies optimistic, a sensitivity analysis repeated the screen inside every replicate (Section 2.15). Selection frequency is the proportion of replicates in which a gene carried a nonzero coefficient. Genes were ordered by frequency and the leading 12 formed the panel. The panel size of 12 was set before selection, and a frequency of at least 0.60 was specified in advance as a per-gene stability standard to be reported, not as an inclusion rule. The panel therefore contains 12 genes although only six met the standard, and criterion 5 (Section 2.14) reports how many genes met it. Benjamini–Hochberg correction was applied to families of biological tests [39].

### 2.8. Model Lock and Leakage Controls

The term fixed refers to the 12-gene list and its ordering after TCGA-based selection, rather than to a gene list specified before discovery. After feature selection, the panel file was hashed and written together with the full model specification. External scoring recomputes that checksum and halts if the panel file or its ordering has changed. The lock also recorded the master seed, the feature-selection settings, the algorithm list and the primary versus stress-test cohort designations. The criteria are reported as prespecified and analysis-locked; the analysis was not registered on a public platform. A checksum verifies file integrity but does not by itself prove that the lock preceded access to outcomes. Model specifications were fixed at the lock, whereas fitted parameters were subsequently estimated in TCGA. External cohort medians and median absolute deviations were estimated without outcomes under the fixed preprocessing rule; their numeric values were not transported from TCGA. No clinical decision threshold was established. Internal cross-validation was carried out after panel selection from the full discovery cohort and is therefore descriptive; it was not used as an unbiased estimate of generalization or to select a model.

### 2.9. Locked Survival Models

All molecular models received the same 12 standardized gene features. The age and sex Cox model received standardized age and a male indicator only. At the time of the lock, pathological stage had not been harmonized across both primary external cohorts, so stage comparators were specified after the lock (Section 2.15). Median age imputation was performed within each cohort. The panel Cox model used L2-regularized Cox regression with alpha 0.1 [37]. The random survival forest used 500 trees, minimum split size 10, minimum leaf size 8, square-root feature sampling and a fixed seed [28]. Gradient boosting survival used the Cox partial-likelihood loss, 300 estimators, learning rate 0.03 and maximum depth 2. DeepSurv used fully connected layers of 32 and 16 units, SiLU activations, dropout 0.15 and a single risk output [27]. AdamW optimization used a learning rate of 0.002, weight decay of 0.001 and gradient-norm clipping at 5, with at most 400 epochs for external fits and early stopping after 40 non-improving epochs of the training partial-likelihood loss. No TCGA patients were held out for early stopping in the locked specification, so a post-lock sensitivity analysis repeated the fit with stopping on a stratified 20% validation split. SurvivalPFN used the released shi-ang/SurvivalPFN checkpoint with in-context fitting on the TCGA training examples [30]. The median predicted event time was multiplied by minus one to define a risk score, so that larger values denote earlier predicted events. SurvivalPFN returns a discretized event-time distribution, and the median was interpolated within the bin in which the cumulative distribution reached 0.5. A patient whose distribution did not reach 0.5 within the finite bins would have received an infinite median and the lowest risk; this did not occur, and every external patient had a finite predicted median. SurvivalPFN and DeepSurv ran on CUDA using an NVIDIA RTX 4090 GPU (NVIDIA Corporation, Santa Clara, CA, USA). CPU models were implemented with scikit-survival [40] and GPU tensor operations with PyTorch [41].

### 2.10. Internal Resampling and Locked External Scoring

CPU models were evaluated with repeated stratified five-fold cross-validation using three repeats. GPU models used stratified five-fold cross-validation. All splits were patient-level and stratified by event status. After the lock, each estimator was fitted on all TCGA discovery patients and scored in the three external cohorts without retraining on external outcomes. Harrell’s concordance index was the primary discrimination measure [42]. One thousand patient-level bootstrap resamples produced 95% confidence intervals for each model and cohort. For direct comparisons, 2000 paired bootstrap resamples used the same resampled patients for SurvivalPFN and its comparator; the reported delta is C-index (SurvivalPFN) minus C-index (comparator), and an interval excluding zero was required to support a difference. Uno’s inverse-probability-of-censoring-weighted C-index and cumulative/dynamic area under the curve at 1, 3 and 5 years were secondary measures [43,44]. A horizon was marked as not estimable where follow-up, censoring or event support did not satisfy the estimator. The number of deaths before each horizon and the number of patients still under observation are reported in Table S1.

### 2.11. Survival-Distribution Calibration

Calibration was evaluated for SurvivalPFN and for panel Cox [24]. TCGA patients were split 70:30 into model-training and calibration subsets, stratified by event status, and SurvivalPFN was fitted in the 70% subset. For each calibration patient, the 0.1 through 0.9 event-time quantiles were obtained, censored times were represented by 200 conditional Kaplan–Meier samples, and the conformal corrections were estimated from the held-out 30% subset [31]. No external outcome contributed to the corrections, and the external patients used to assess calibration were distinct from the TCGA patients used to estimate them. This analysis used the 70% TCGA fit and should be distinguished from primary discrimination scoring after fitting on all TCGA patients. Target-cohort recalibration was not performed. Raw and conformalized survival distributions were evaluated on a 30-point time grid bounded by cohort follow-up and a maximum of five years. The integrated Brier score, 1- and 3-year Brier scores and D-calibration were computed [31,45]. A lower integrated Brier score indicates more accurate probabilistic prediction. D-calibration tests whether predicted survival probabilities at observed event times follow their expected distribution, with censoring contributions distributed across compatible bins. Because a non-significant D-calibration test does not by itself establish calibration, the Brier direction and the D-calibration result were interpreted jointly.

The 30% calibration subset contained 152 patients and 55 deaths, which limits the precision of the corrections. For panel Cox, survival probabilities were derived from the Breslow baseline hazard estimated in TCGA. For both models, the integrated Brier score was reported with 1000-replicate patient-level bootstrap intervals and compared with a covariate-free Kaplan–Meier reference estimated in each target cohort. The index of prediction accuracy was calculated as one minus the ratio of the model score to the reference score [46]. The calibration slope was the coefficient of the transported prognostic index in a Cox model fitted in the target cohort, using the linear predictor for panel Cox and the complementary log-log of predicted 3-year risk for SurvivalPFN. A slope of 1 indicates ideal spread. Calibration-in-the-large was summarized as the ratio of observed (Kaplan–Meier) to mean predicted 3-year risk, and grouped calibration was plotted by quintile of predicted risk.

### 2.12. Effect-Direction Transport

For each selected gene, an L2-regularized univariable Cox model was fitted independently in TCGA and in each external cohort, and coefficient signs were compared with the TCGA sign. The prespecified direction criterion required at least 75% agreement across the two primary cohorts when all gene-cohort comparisons were aggregated. GSE31210 direction was recorded as a stress-test observation. These coefficients are not adjusted effects and were not used to revise the panel. Because a sign comparison treats a coefficient near zero in the same way as a large one, univariable hazard ratios per robust standard-deviation unit, with 95% confidence intervals, were also estimated in every cohort with unpenalized Cox models.

### 2.13. Post-Selection Biological Evaluation

Biological analyses were performed after the model, and the external predictions were locked. The all-sample TCGA variance-stabilized matrix was aligned row for row to exact GENCODE identifiers. Patients contributing both a selected primary tumor and a solid-tissue normal sample formed the paired set. For each panel gene, the paired tumor-minus-normal change was tested with a two-sided Wilcoxon signed-rank test, paired Cohen’s *d*_z_ summarized the standardized within-patient change, and the false discovery rate was controlled across the panel [39]. Stage association was assessed among the 496 patients with TCGA-CDR stage I–IV information by correlating ordered stage with expression using Spearman’s rho, with false-discovery-rate correction across the panel. Functional enrichment queried g:Profiler for Gene Ontology, Reactome, KEGG, WikiPathways and CORUM resources [47], and protein associations were queried through STRING v12 at medium confidence 0.4 [48]. API responses were cached. Because the panel could only be drawn from the 1232-gene selection universe, the enrichment analysis was repeated with that universe as a custom background under both g:SCS and Benjamini–Hochberg correction. It was complemented by hypergeometric tests against MSigDB Hallmark, Reactome and KEGG Medicus gene sets restricted to the same universe. These analyses characterize expression; they do not establish mechanism, independence from clinicopathological variables or therapeutic actionability, and they could not alter the prespecified criteria.

### 2.14. Prespecified Interpretation Criteria

A positive biomarker interpretation required all six of the following, applied conjunctively: (1) the SurvivalPFN point estimate exceeds the age and sex Cox model in both primary cohorts; (2) at least one primary paired SurvivalPFN-versus-age-and-sex interval excludes zero; (3) conformalization preserves or improves the integrated Brier score within a tolerance of 0.005 in both primary cohorts; (4) aggregate primary-cohort direction agreement is at least 75%; (5) every panel gene reaches a selection frequency of at least 0.60; and (6) the frozen model is scored in the early-stage cohort. Any unmet criterion required a comparative-methods interpretation without claiming algorithmic superiority, clinical readiness or successful validation against the complete six-criterion standard. Criterion 6 is procedural, being completed once the frozen model is scored in GSE31210, and is therefore reported separately from the five substantive criteria (1 to 5).

### 2.15. Post-Lock Analyses

Age and sex were concatenated with the 12 genes for SurvivalPFN. XGBoost (version 3.4.1) with a Cox objective used 600 trees, depth 2, learning rate 0.025, GPU execution and fixed seeds [32]. Stage-only Cox models form part of the stage benchmark described below. All three analyses were specified after the primary lock.

Stage benchmark. TCGA-CDR stage was grouped as I to IV. GSE72094 reported stage groups directly, and GSE31210 reported stage I or II. GSE68465 reported pathological T and N categories, which were grouped according to the sixth edition of the AJCC system, with M0 assumed for these resected patients: pT1-2N0 as stage I, pT1-2N1 or pT3N0 as stage II, and all other combinations as stage III. Stage-only, panel and stage-plus-panel Cox models, and the same models with age and sex, were fitted in TCGA with ordinal stage and an L2 penalty of 0.1 and scored unchanged. Patients without stage (8 in TCGA, 5 in GSE72094 and 3 in GSE68465) were excluded from these comparisons. Paired differences used 2000 shared bootstrap resamples. Within each external cohort, Cox models with categorical stage, with or without age and sex, were extended by the standardized locked panel score or SurvivalPFN score, and the added information was tested with a likelihood-ratio test. Panel discrimination was also estimated within stage I, and patients were assigned to low-, intermediate- and high-risk groups using TCGA tertiles of the panel Cox score.

Random-signature reference and model assumptions. One thousand 12-gene panels were drawn at random from the 1232-gene universe, fitted with the panel Cox specification in TCGA and scored in each external cohort [21]. The empirical *p* value was the proportion of random panels with a C-index at least as high as that of the locked panel. Proportional hazards were assessed with scaled Schoenfeld residuals against rank-transformed time [49] for the 12-gene model in TCGA and for the panel score and stage in every cohort. Median follow-up was estimated with the reverse Kaplan–Meier method [50].

Panel and training sensitivity. The six genes meeting the 0.60 standard were refitted with each learner. Each gene’s median expression was ranked among all genes measured on the same platform, and genes in the bottom 10% on any microarray (or, more strictly, the bottom 25%) were removed before refitting panel Cox. The bootstrap was repeated with the univariable screen inside each replicate, and DeepSurv was refitted with validation-based early stopping. Panel Cox was refitted after excluding the 17 patients whose flattened diagnosis field carried a non-adenocarcinoma label and, separately, with TCGA-CDR survival.

Cohort overlap. Each GSE68465 patient was compared with every GSE72094 patient for identical clinical records (same sex, age within 1 year, same stage group, same vital status and survival within 31 days). Expression similarity was also compared across the 1232 shared genes, expressed as the best-match correlation standardized against that patient’s correlations with all GSE72094 samples. Patients from the three GSE68465 sites other than Moffitt cannot appear in GSE72094 and served as the null reference. GSE68465 was also re-evaluated after excluding the Moffitt subset. All analyses in this section were performed after the lock, and none changed the panel, the locked models or the prespecified criteria.

### 2.16. Statistical Analysis, Reporting and Software Environment

All statistical tests were two-sided, and the alpha level was set at 0.05. The paired tumor-normal comparison used the Wilcoxon signed-rank test and the stage-trend analysis used Spearman’s rank correlation; both are distribution-free and were chosen because the variance-stabilized expression values were not assumed to follow a normal distribution. Multiplicity across the 12-gene panel was controlled with the Benjamini–Hochberg procedure within each family of tests, and false discovery rates are reported alongside the corresponding statistics [39]. Uncertainty for concordance indices and for their paired differences was obtained by patient-level bootstrap resampling rather than by a parametric test, which avoids distributional assumptions on the concordance index. We used 1000 resamples for marginal intervals and 2000 paired resamples for model comparisons. All *p* values are reported as computed rather than as threshold statements, and descriptive statistics are given with the cohort size and event count that produced them. External validation of a survival model is generally recommended to include at least 100 events [51,52]. GSE31210, with 35 deaths, falls short of this recommendation and was therefore prespecified as an early-stage stress-test cohort rather than a primary validation cohort. RNA-seq normalization used R 4.6.0 with DESeq2 1.52.0. All remaining stages ran under Python 3.11.15. Post-lock analyses ran under Python 3.12 with scikit-survival 0.28.0, scikit-learn 1.9.1, lifelines 0.30.3, NumPy 2.5.3, pandas 2.3.3, SciPy 1.18.1 and PyTorch 2.14.1 on CPU, with the released SurvivalPFN v0.1 checkpoint. Refitting panel Cox and SurvivalPFN in this environment reproduced the locked external C-indices exactly. Reporting follows TRIPOD and its artificial-intelligence extension [53,54], and risk of bias was assessed against PROBAST+AI [55]. Performance is reported with uncertainty and calibration, consistent with recommendations for external validation [56,57,58].

## 3. Results

### 3.1. Cohort Construction and Preprocessing

**Figure 1:**
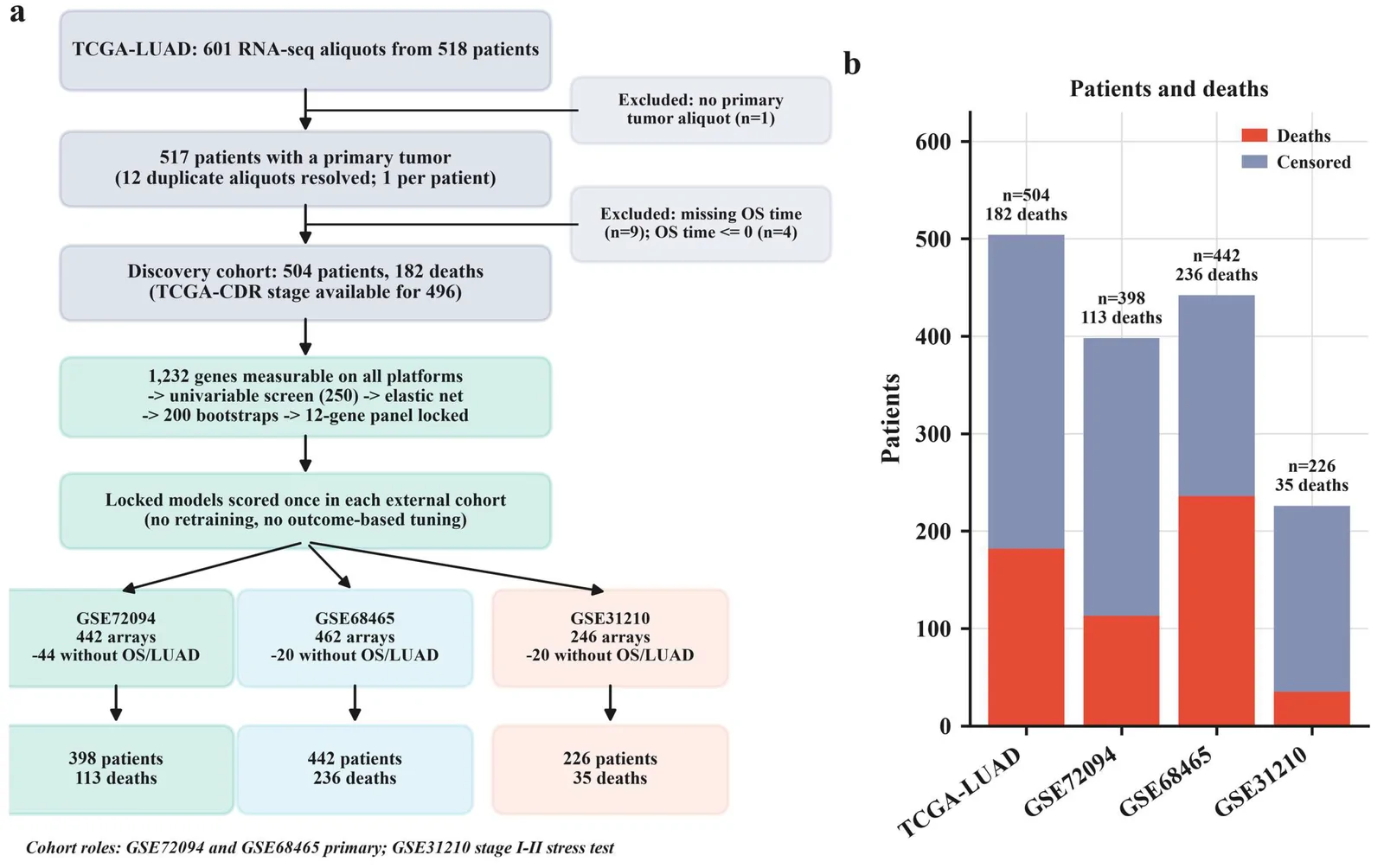
Study flow and analysis lock. (**a**) Patient flow from raw TCGA aliquots to the 504-patient discovery cohort, panel selection and lock, and from the GEO series to the three external cohorts, with every exclusion and its count. (**b**) Deaths and censored patients in the discovery cohort and each validation cohort.

The input audit identified 601 TCGA aliquots from 518 patients. One patient had no primary-tumor aliquot, leaving 517. Deterministic resolution of duplicate primary tumors and exclusion of 9 patients without survival time and 4 with non-positive survival time produced 504 discovery patients with 182 deaths (Figure 1a). Count filtering retained 26,356 genes, and exact GENCODE mapping covered all 60,660 input rows with no unmatched identifiers. Outcome-blind variance selection produced a 504 × 2000 protein-coding discovery matrix, of which 1232 genes were measurable on all three external platforms and formed the selection universe. TCGA-CDR stage was available for 496 patients, including 390 with stage I–II disease, of whom 119 died. External evaluation covered 1066 patients and 384 deaths after exclusion of 44, 20 and 20 arrays without eligible survival annotation; in GSE31210, the excluded arrays were non-tumor lung samples (Figure 1, Table 1). Event counts and follow-up differed substantially between cohorts, and this heterogeneity is what makes the set informative about transport. GSE68465 supplied the largest number of events and GSE31210 the smallest, with 35 deaths among 226 stage I–II patients. Median follow-up by the reverse Kaplan–Meier method was 2.4 years (95% CI 2.2–2.7) in TCGA, 2.5 years (2.4–2.6) in GSE72094, 6.3 years (6.0–6.9) in GSE68465 and 5.1 years (4.9–5.2) in GSE31210 (Table 1, Figure S1). In GSE72094, only 68 patients remained under observation beyond 3 years and 5 beyond 5 years.

Count filtering and the protein-coding restriction reduced 60,660 input genes to 16,888 symbols, and the outcome-blind variance filter reduced this to 2000 (Figure S2). Platform coverage of that set was 1802 symbols in GSE72094, 1288 in GSE68465 and 1833 in GSE31210. GSE68465 was the limiting platform, and the intersection across all four cohorts defined the 1232-gene selection universe.

### 3.2. Panel Selection Stability and Effect-Direction Transport

**Figure 2:**
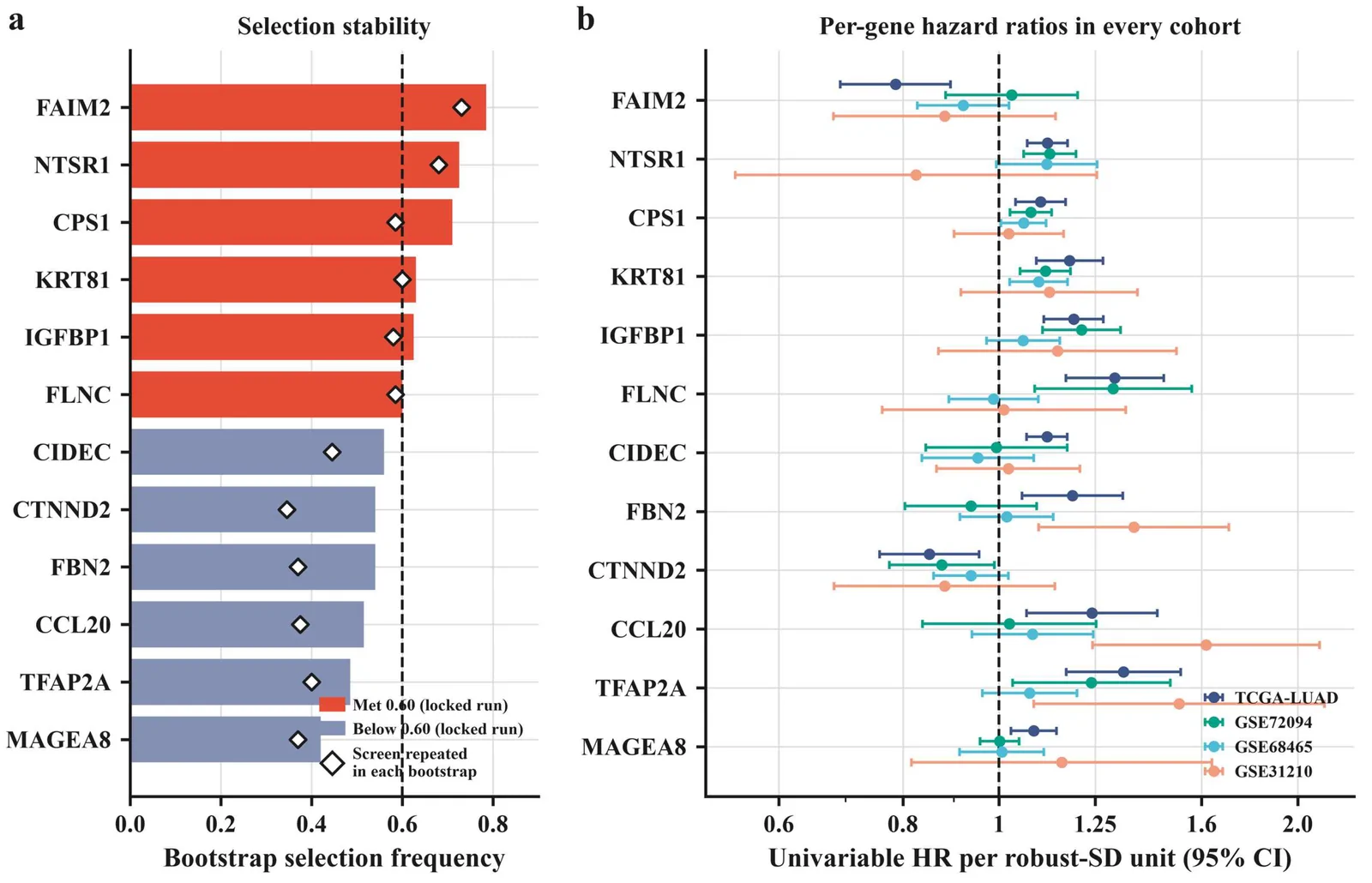
Panel stability and gene-level transport. (**a**) Bootstrap selection frequency for the 12 panel genes. The dashed line marks the prespecified 0.60 threshold; red bars denote genes that met it in the locked selection and blue bars those that did not. Open diamonds show frequencies when the univariable screen was repeated inside each bootstrap replicate. (**b**) Univariable hazard ratios per robust standard-deviation unit with 95% confidence intervals for each gene in the discovery cohort and each external cohort; the dashed line marks a hazard ratio of 1.

Selection frequency ranged from 0.785 for *FAIM2* to 0.420 for *MAGEA8* (Figure 2a, Table S2). *FAIM2*, *NTSR1*, *CPS1*, *KRT81*, *IGFBP1* and *FLNC* met the prespecified 0.60 standard, and *CIDEC*, *FBN2*, *CTNND2*, *CCL20*, *TFAP2A* and *MAGEA8* fell below it. Frequencies declined gradually rather than dropping sharply after the sixth gene, the pattern expected when correlated candidates compete for selection. Selection frequency tracked effect magnitude: the six genes above the threshold carried the largest median absolute coefficients, with *FAIM2* highest on both measures (Figure S3a). When the univariable screen was repeated inside each bootstrap replicate, frequencies fell by 0.04 to 0.20, but 10 of the 12 panel genes remained among the 12 most frequently selected and all 12 among the top 20. *FAIM2* (0.730), *NTSR1* (0.680) and *KRT81* (0.600) still met the 0.60 standard (Figure 2a, Table S3). Across GSE72094 and GSE68465, 79.2% of gene-cohort coefficient signs agreed with TCGA, meeting the aggregate direction criterion. Agreement was 75% in GSE72094, 83% in GSE68465 and 92% in the early-stage cohort (Figure S4, Table S4). Because a sign can agree or disagree by chance when an effect is close to zero, hazard ratios with confidence intervals were also estimated for every gene in every cohort (Figure 2b, Table S5). Of the 36 external estimates, 12 had intervals excluding 1, all in the same direction as in TCGA: 7 in GSE72094, 2 in GSE68465 and 3 in GSE31210. Every sign reversal (*FAIM2*, *CIDEC* and *FBN2* in GSE72094, *FLNC* and *CIDEC* in GSE68465, and *NTSR1* in GSE31210) had an interval spanning 1. Seven genes agreed in all three external cohorts: *CPS1*, *KRT81*, *IGFBP1*, *CTNND2*, *CCL20*, *TFAP2A* and *MAGEA8*. By this sign-based measure, the genes below the stability threshold transported at least as consistently as the stable genes: four of the six less stable genes agreed in every cohort, against three of the six stable genes. By effect size, the picture was more balanced. The stable genes contributed 7 of the 12 external hazard ratios whose intervals excluded 1 (*CPS1* and *KRT81* in both primary cohorts, and *NTSR1*, *IGFBP1* and *FLNC* in GSE72094), and the less stable genes contributed 5. Transport was therefore not confined to the most stable genes, which supports retaining the full panel. *CIDEC* transported least consistently, agreeing in 1 of 3 cohorts. *FAIM2* reversed sign only in GSE72094, where its hazard ratio was 1.03 (95% CI 0.88–1.20). Because each model is univariable and platform-specific, agreement indicates transport of the direction of association rather than an independent prognostic contribution.

### 3.3. Discrimination in the Locked External Cohorts

**Figure 3:**
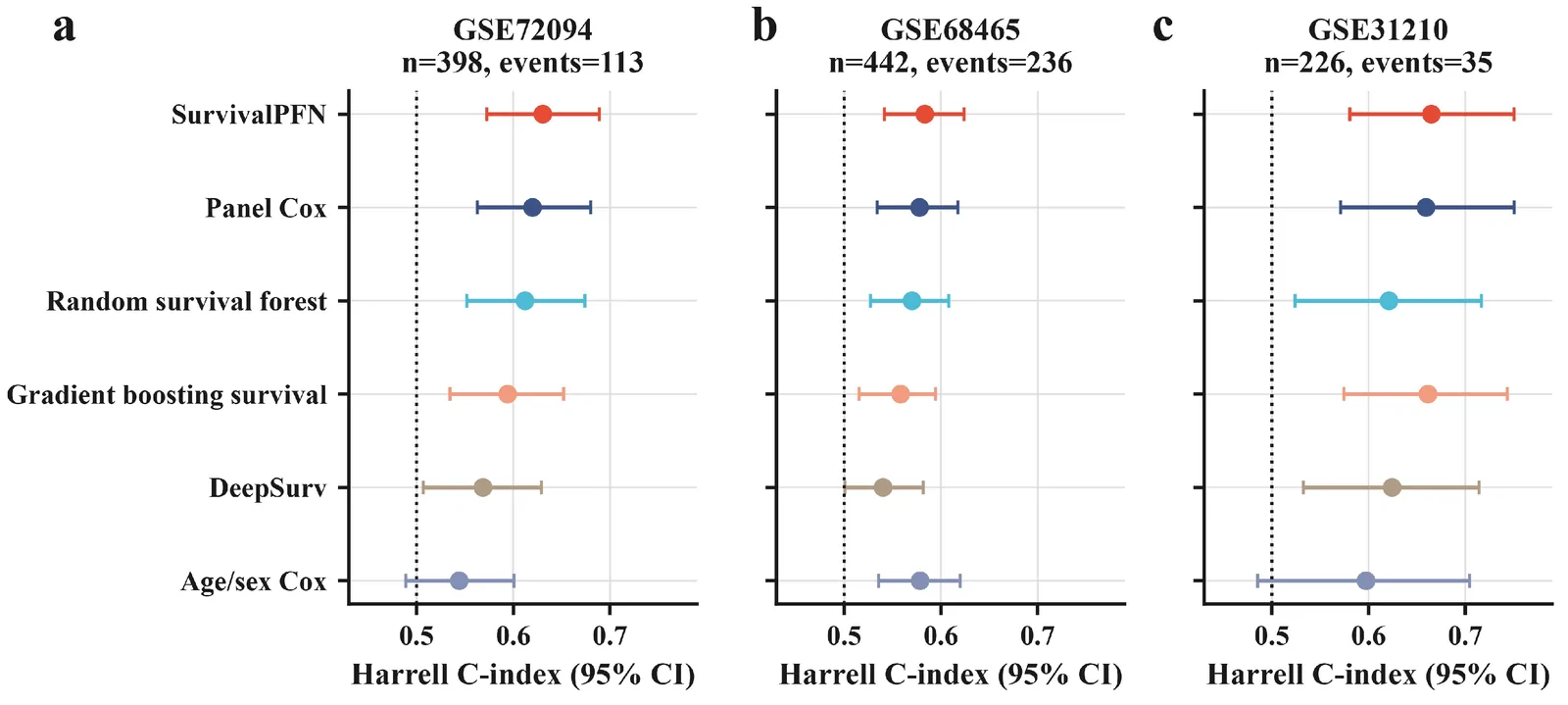
External discrimination after the model lock. Points are Harrell C-indices and horizontal bars are 95% patient-level bootstrap confidence intervals for (**a**) GSE72094, (**b**) GSE68465 and (**c**) GSE31210. All models share the same 12 molecular predictors except the age and sex Cox comparator.

Internal resampling gave mean C-indices of 0.682 for SurvivalPFN, 0.675 for panel Cox, 0.673 for random survival forest, 0.663 for gradient boosting, 0.642 for DeepSurv and 0.501 for the age and sex model (Figure S3b, Table S6). These values are descriptive because the panel was selected before this resampling, and the external cohorts carry the inference. In the locked external evaluation, SurvivalPFN gave the highest Harrell C-index in all three cohorts: 0.631 (95% CI 0.572–0.689) in GSE72094, 0.583 (95% CI 0.541–0.624) in GSE68465 and 0.665 (95% CI 0.581–0.750) in GSE31210 (Figure 3, Table 2). Panel Cox gave 0.620, 0.578 and 0.659, so SurvivalPFN and a conventional model built on the same genes were within 0.005 to 0.011 of each other. Confidence intervals overlapped among the molecular learners, most widely in the low-event early-stage cohort. Below SurvivalPFN, the ranking varied: gradient boosting (0.661) narrowly exceeded panel Cox (0.659) in GSE31210, where random survival forest (0.621) had the lowest molecular-model estimate. The age and sex comparator varied by cohort: 0.544 in GSE72094, below every molecular model, and 0.578 in GSE68465, equal to panel Cox. In practical terms, a C-index of 0.63 means that in 63 of every 100 comparable pairs of patients, the patient who died first had the higher predicted risk, against 50 expected by chance. On an absolute scale, TCGA-derived tertiles of the panel Cox score separated the low- and high-risk groups in every cohort. Three-year overall survival in these two groups was 69.9% and 51.3% in GSE72094 (hazard ratio 2.39, 95% CI 1.45–3.94), 77.1% and 60.3% in GSE68465 (1.43, 1.02–2.01) and 94.8% and 82.1% in GSE31210 (3.65, 1.66–8.03) (Figure S5, Table S7).

### 3.4. Paired Model Comparisons

**Figure 4:**
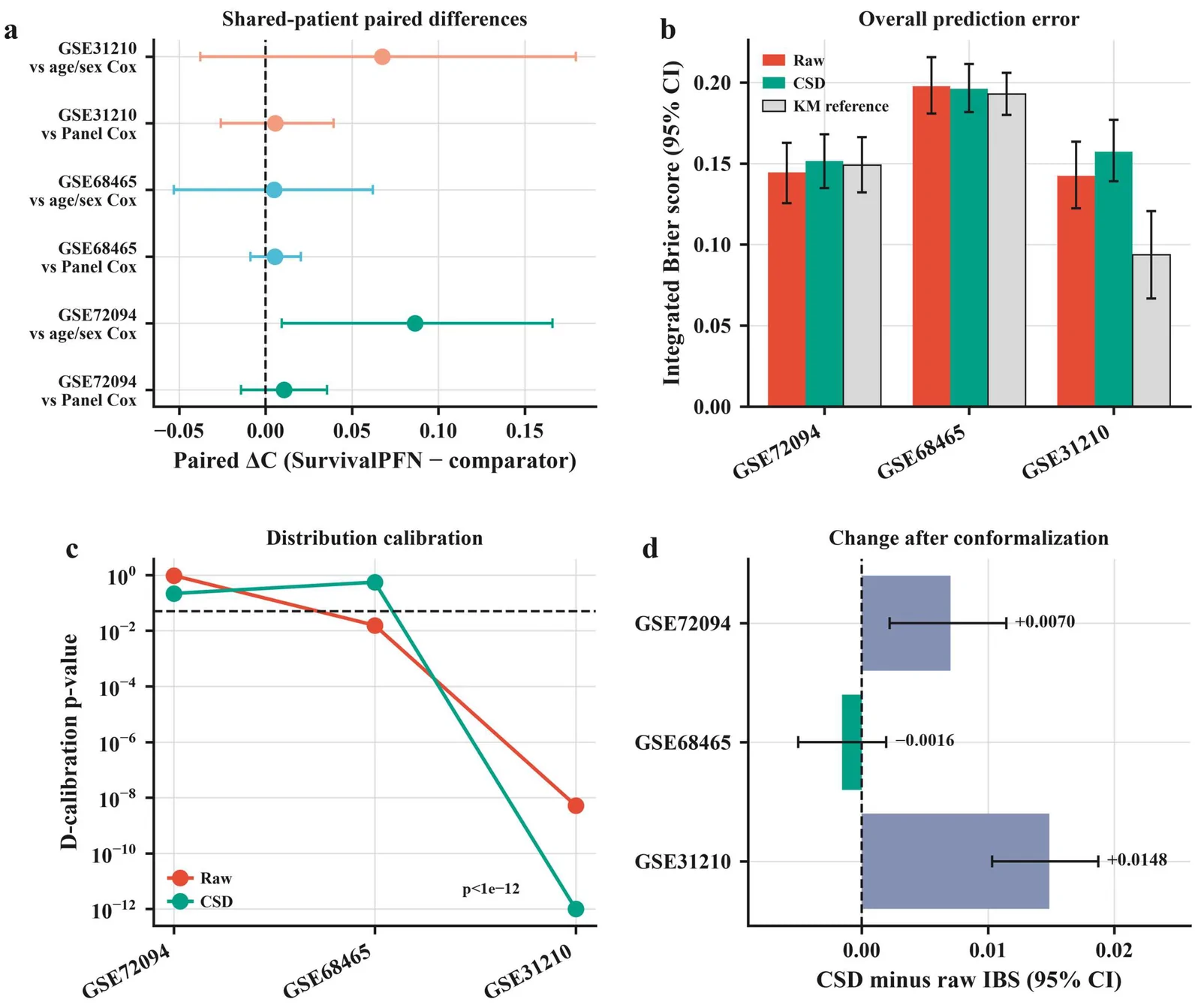
Paired uncertainty and calibration transport. (**a**) Paired bootstrap ΔC for SurvivalPFN against the age and sex Cox model and against panel Cox in each cohort; the dashed line marks no difference. (**b**) Integrated Brier score for raw and conformalized predictions and for a covariate-free Kaplan–Meier reference, with 95% bootstrap confidence intervals. (**c**) D-calibration *p* values on a logarithmic scale, with the dashed line at *p* = 0.05. (**d**) Change in integrated Brier score after conformalization, with 95% bootstrap confidence intervals, where negative values indicate improvement.

Models were compared on identical resampled patients (Figure 4a, Table S8). Against the age and sex model, the SurvivalPFN ΔC was 0.086 (95% CI 0.009–0.166) in GSE72094, 0.005 (95% CI −0.053 to 0.062) in GSE68465 and 0.068 (95% CI −0.038 to 0.179) in GSE31210. The GSE72094 interval excluded zero, satisfying the prespecified uncertainty criterion. Against panel Cox, ΔC was 0.011 (95% CI −0.014 to 0.036), 0.005 (95% CI −0.009 to 0.020) and 0.006 (95% CI −0.026 to 0.039); all three intervals included zero. These results do not establish a difference, formal equivalence or non-inferiority. In summary, SurvivalPFN gave the highest point estimate in every cohort, exceeded the clinical comparator with an interval excluding zero in one cohort, and showed only small point-estimate gains over regularized Cox regression given the same 12 genes. All paired differences, including the comparisons with stage reported in Section 3.5 and with the six-gene core, are collected in Table 3.

### 3.5. Prognostic Value Beyond Pathological Stage

**Figure 5:**
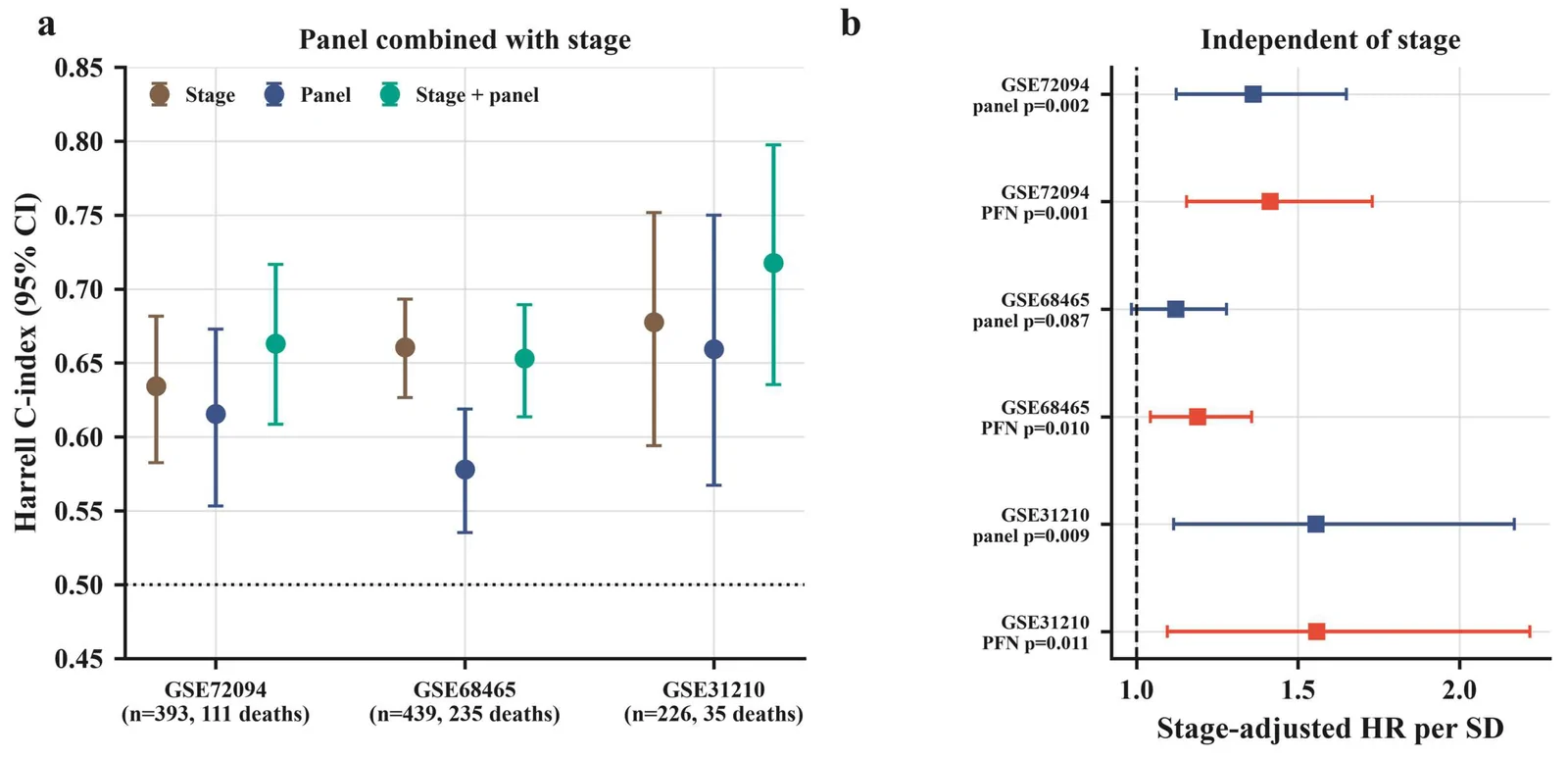
Prognostic value of the panel beyond pathological stage. (**a**) Harrell C-index with 95% bootstrap confidence intervals for stage-only, panel and combined stage-plus-panel Cox models fitted in TCGA and scored unchanged in patients with recorded stage. (**b**) Hazard ratio per standard deviation of the panel Cox score (blue) and the SurvivalPFN score (red) after adjustment for stage within each cohort, with likelihood-ratio *p* values.

The panel score was associated with survival independently of pathological stage. Per standard deviation, the stage-adjusted hazard ratio was 1.36 (95% CI 1.12–1.65; likelihood-ratio *p* = 0.002) in GSE72094, 1.56 (1.11–2.17; *p* = 0.009) in GSE31210 and 1.12 (0.98–1.28; *p* = 0.087) in GSE68465. These estimates were essentially unchanged after further adjustment for age and sex (Figure 5b, Table 4 and Table S9). The SurvivalPFN score was independently associated with survival in all three cohorts, with stage-adjusted hazard ratios of 1.41, 1.19 and 1.56 (all *p* ≤ 0.011). Within stage I, the panel stratified patients with C-indices of 0.607 (0.525–0.688), 0.556 (0.498–0.615) and 0.703 (0.566–0.822), separating risk among patients whom stage places in the same group.

Stage alone set a demanding benchmark. Stage-only Cox gave C-indices of 0.634, 0.661 and 0.678, at or above those of the gene-only models in every cohort, and neither the panel Cox score nor the SurvivalPFN score outperformed stage in any cohort (Table 3). Combining the panel with stage gave the highest discrimination of any model in GSE72094 and GSE31210, with C-indices of 0.663 (95% CI 0.608–0.717) and 0.718 (0.635–0.797), compared with 0.634 (0.582–0.682) and 0.678 (0.594–0.752) for stage alone (Figure 5a, Table 4). These gains of 0.029 (−0.024 to 0.081) and 0.040 (−0.062 to 0.135) did not reach statistical significance (Table 3). In GSE68465, stage alone gave 0.661 (0.627–0.693) and the combined model 0.653 (0.613–0.689). The panel is therefore best used together with stage rather than in its place.

### 3.6. Calibration Transport

Both locked models reproduced average absolute risk in the primary cohorts. The ratios of observed to predicted 3-year risk were 1.02 and 1.00 for panel Cox and 1.10 and 1.08 for raw SurvivalPFN in GSE72094 and GSE68465. Panel Cox reduced the integrated Brier score of a covariate-free Kaplan–Meier reference by 3.8% in GSE72094 (Figure S6, Table S10). Calibration slopes were 0.56, 0.28 and 0.78 for panel Cox and 0.51, 0.29 and 0.79 for SurvivalPFN, indicating that predicted risks were more widely spread than observed outcomes. In early-stage GSE31210, observed 3-year risk was 9.1% against a mean panel Cox prediction of 33.4% (ratio 0.27); stage-only Cox gave a similar ratio (0.31), consistent with the lower baseline hazard of this cohort.

Conformalization had cohort-dependent effects on probabilistic prediction (Figure 4b–d, Table S11). In GSE68465, the integrated Brier score improved from 0.19767 to 0.19611 (difference −0.0016, 95% CI −0.0050 to 0.0019) and the D-calibration *p* value increased from 0.016 to 0.556, reflecting a reduced excess in the first and last bins. In GSE72094, the integrated Brier score increased from 0.14450 to 0.15153 (+0.0070, 0.0022 to 0.0114), and in GSE31210 from 0.14245 to 0.15730 (+0.0148, 0.0103 to 0.0187); one-year Brier scores were also higher after correction in all three cohorts. Thus, a correction learned in TCGA did not consistently improve external probabilistic accuracy. D-calibration histograms are shown in Figure S7, and the raw GSE72094 test did not reject calibration (*p* = 0.96).

### 3.7. Time-Dependent Discrimination

Uno’s C-index preserved the primary ranking, with SurvivalPFN at 0.609 in GSE72094, 0.579 in GSE68465 and 0.653 in GSE31210 (Figure S8, Table S12). Cumulative/dynamic area under the curve gave no single consistent ordering. Panel Cox was higher at one year in GSE68465 (0.6614 against 0.6609) and at three years in GSE31210 (0.684 against 0.673), where the age and sex model also reached 0.684. Late horizons at which risk sets and censoring support were inadequate are labeled NE, and no missing performance value was imputed. Model ranking is therefore conditional on the estimand, and a model that ranks well across all observed times need not be best at a single horizon. Horizon support differed sharply between cohorts (Table S1). GSE72094 had 43 deaths before 1 year and 106 before 3 years, but only 68 patients remained under observation beyond 3 years, so its Uno truncation time of 1036 days fell before the 3-year horizon. GSE31210 had 4, 20 and 32 deaths before 1, 3 and 5 years, and GSE68465 had 48, 140 and 187.

### 3.8. Matched Tumor-Normal Expression and Functional Annotation

**Figure 6:**
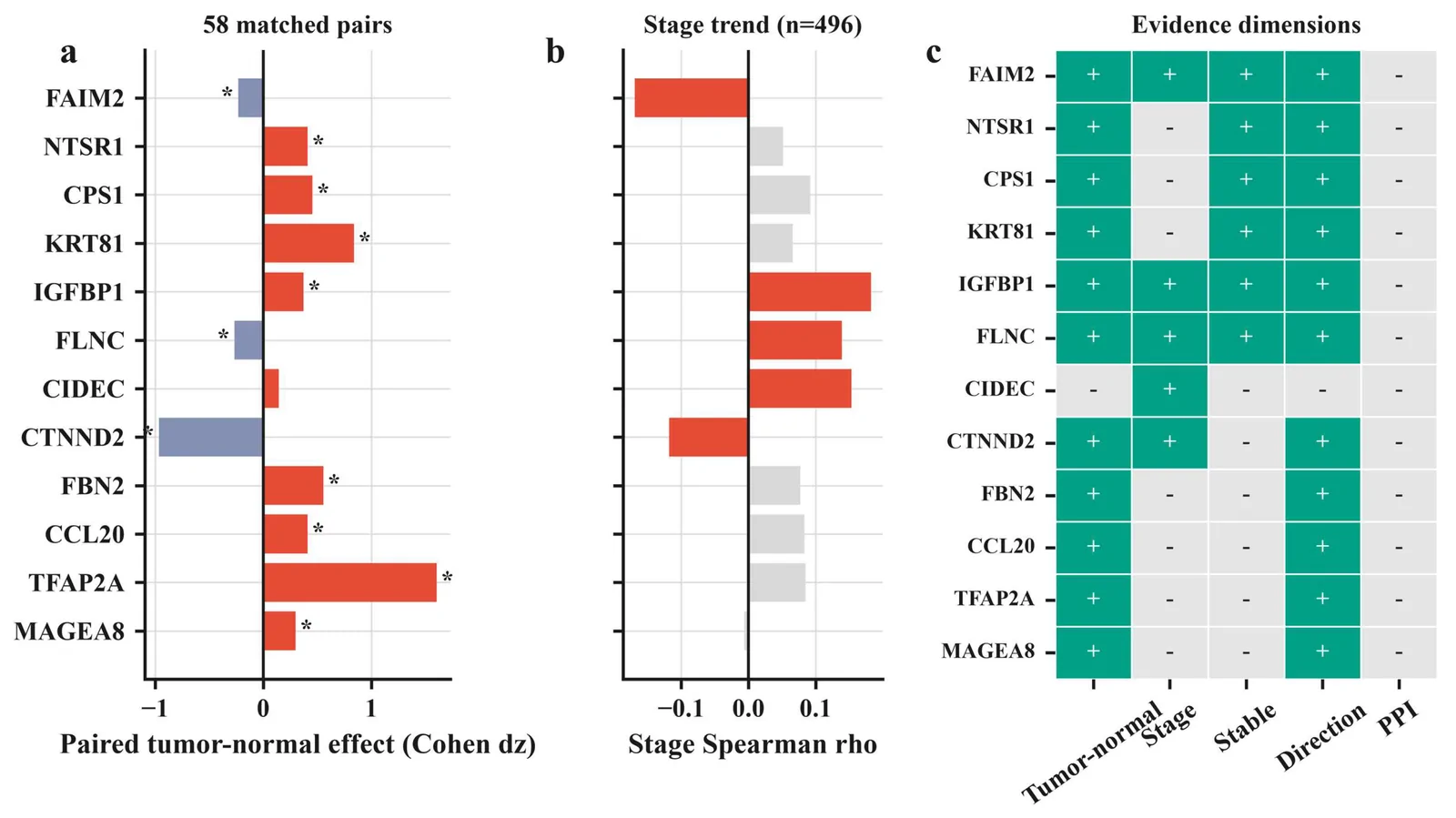
Biological evaluation of the selected transcripts. (**a**) Paired tumor-minus-normal effect sizes in 58 matched pairs, where asterisks denote a false discovery rate below 0.05. (**b**) Spearman correlation between expression and ordered pathological stage in 496 patients with TCGA-CDR stage, with red marking genes that passed correction. (**c**) Evidence matrix separating tissue dysregulation, stage trend, selection stability, survival-direction transport and protein-interaction support for each gene.

Fifty-eight patients contributed both a selected primary tumor and a solid-tissue normal sample, and 11 of the 12 panel genes differed between the two at a false discovery rate below 0.05 (Figure 6a, Table 5 and Table S13). *TFAP2A* showed the largest standardized increase, with a paired Cohen’s *d*_z_ of 1.60 and a median within-patient rise of 2.79 on the variance-stabilized scale, followed by *KRT81* at *d*_z_ = 0.84. *CTNND2* showed the largest decrease at *d*_z_ = −0.97, with a median within-patient fall of 2.84. *CIDEC* was the single gene that did not differ (false discovery rate 0.967), and it was also the gene whose effect direction was transported least consistently. Because each comparison is made within a patient, these tests control for patient-level factors that confound an unmatched tumor-versus-normal contrast. Stage associations, reassessed in the 496 patients with TCGA-CDR stage, reached the false-discovery threshold for five genes: *FAIM2* (rho −0.169), *IGFBP1* (0.182), *CIDEC* (0.153), *FLNC* (0.139) and *CTNND2* (−0.118) (Figure 6b, Table S14). No Gene Ontology, Reactome, KEGG, WikiPathways or CORUM term reached significance across the queried databases (Table S15), and STRING returned no medium-confidence interactions among the 12 proteins, with a protein–protein interaction enrichment *p* value of 1.0 (Table S16). Restricting the background to the 1232-gene selection universe did not change this picture. No term reached significance under g:SCS correction; only two CORUM complexes, each with a single annotated gene in the universe, passed Benjamini–Hochberg correction, and no Hallmark, Reactome or KEGG Medicus set was enriched (Table S17). The panel therefore aggregates genes with individual tumor-associated expression changes rather than measuring one pathway or a physically interacting module.

### 3.9. Outcome of the Prespecified Interpretation Criteria

Three of the five substantive criteria were met, and the procedural sixth criterion was completed (Figure S9, Table S18). The SurvivalPFN point estimate exceeded the age and sex model in both primary cohorts, the paired interval excluded zero in one primary comparison against that model, and aggregate direction concordance reached 79.2% against a 75% requirement. The two unmet criteria concerned calibration and stability: conformalization preserved the integrated Brier score in only one of the two primary cohorts, and only 6 of the 12 genes reached the stability threshold. Because the criteria were applied conjunctively, the study is reported as an externally evaluated comparative study.

### 3.10. Post-Lock Sensitivity Analyses

Adding age and sex to SurvivalPFN gave C-indices of 0.635, 0.595 and 0.667 in GSE72094, GSE68465 and GSE31210, a gain of 0.004 to 0.012 over the locked molecular model, which was largest where the clinical comparator was strongest (Figure S10, Table S19). XGBoost Cox gave 0.566, 0.556 and 0.634, below SurvivalPFN and panel Cox in all three cohorts. Stage comparisons are reported in Section 3.5. Because these analyses were specified after the lock, they are hypothesis-generating and do not count for or against the prespecified criteria.

The full 12-gene panel was needed to preserve discrimination in early-stage disease. The six stable genes performed comparably in the primary cohorts, with panel Cox C-indices of 0.621 and 0.572 and paired differences from the 12-gene panel of 0.001 (95% CI −0.036 to 0.039) and −0.005 (−0.038 to 0.028). In GSE31210, by contrast, the 12-gene panel exceeded its six-gene core by 0.104 (0.036–0.179) with panel Cox and by 0.115 (0.051–0.184) with SurvivalPFN (Table 3 and Table S20). Results were insensitive to weakly expressed genes. Removing the four genes with median expression in the bottom 10% of any microarray (*IGFBP1*, *KRT81*, *MAGEA8* and *NTSR1*) changed the panel Cox C-index by only 0.010 to 0.013 (eight-gene C-indices 0.607, 0.566 and 0.650; Figure S11), whereas a stricter bottom-quartile rule that kept only three genes gave 0.588, 0.519 and 0.590. Among 1000 random 12-gene panels drawn from the same universe, the locked panel ranked at the 73rd, 70th and 90th percentiles of external C-index (Figure S12, Table S21).

The transported signal did not depend on a single learner. DeepSurv refitted with early stopping on a held-out validation split reached 0.638 (95% CI 0.581–0.692), 0.591 (0.550–0.630) and 0.643 (0.544–0.745), comparable to SurvivalPFN, whereas the locked training-loss rule had given 0.569, 0.540 and 0.624. Excluding the 17 patients with non-adenocarcinoma labels in the flattened diagnosis field changed the external panel Cox C-indices by at most 0.004, and *KRT81* remained associated with survival in TCGA (hazard ratio 1.18). Refitting with TCGA-CDR survival changed the external C-indices by less than 0.001 (Table S20). Proportional hazards were not rejected for the 12-gene model in TCGA (global *p* = 0.42) or for the panel score in GSE72094 and GSE31210 (*p* = 0.055 and 0.59). In GSE68465 (*p* = 0.004), the separation between risk groups was greatest in the early years of follow-up (Figure S5), and stage was non-proportional in GSE31210 (*p* = 0.003) (Table S22).

Results were robust to possible patient overlap between GSE72094 and GSE68465. Three of the 79 Moffitt patients in GSE68465 had a clinically identical record in GSE72094, compared with 26 of 363 patients from the other sites, which cannot overlap. No Moffitt sample showed outlying expression similarity to a GSE72094 sample (largest standardized best-match similarity 5.3, against 5.5 at the other sites), and none of the clinical matches coincided with a top-0.1% expression correlation. Excluding the Moffitt subset left GSE68465 results essentially unchanged: SurvivalPFN 0.595 (95% CI 0.551–0.639), panel Cox 0.588 (0.543–0.633) and age and sex 0.581 (0.531–0.631), with a SurvivalPFN-minus-panel-Cox difference of 0.007 (−0.010 to 0.025) (Table S20).

## 4. Discussion

A 12-gene panel selected in TCGA RNA sequencing data and fixed by checksum ranked patients by overall-survival risk in three independent microarray cohorts accrued on two continents over approximately two decades. Its score remained associated with survival after adjustment for pathological stage in GSE72094 and GSE31210, and the SurvivalPFN score did so in all three cohorts. The panel did not, however, outperform stage: stage-only models discriminated at least as well as the gene-only models in every cohort, and although combining the panel with stage gave the highest discrimination observed in two cohorts, the gain over stage alone did not reach statistical significance. The panel is therefore positioned as a complement to stage rather than a substitute for it. Regularized Cox regression on the same genes performed as well as more complex learners, which indicates that the transported signal resides in the genes rather than in a particular algorithm. Average absolute risk was reproduced in both primary cohorts.

Few published lung cancer signatures have shown prognostic information beyond standard clinical predictors [20], and random gene sets can be associated with outcome [21]. Against this background, a locked panel whose score keeps a stage-adjusted association after transfer from RNA sequencing to microarrays is a meaningful result, even though the panel does not exceed stage on its own. Prognostic expression in LUAD is carried by broad programs, such as proliferation and differentiation, that many genes reflect. This explains why random signatures from a variance-filtered universe also carry signal, and why the locked panel ranked in the upper part of that distribution rather than far outside it. The C-indices of 0.58 to 0.67 mean that the panel ordered risk correctly in roughly 6 of every 10 comparable patient pairs, and its tertiles separated 3-year survival by 12.7 to 18.6 percentage points. The Director’s Challenge established both the value of multi-site blinded validation and the standard that clinical covariates set [10]. Recent signatures have been built from tumor-microenvironment genes, ferroptosis regulators, immune-related RNA-binding proteins and immune programs [15,16,17,18], and a meta-analysis reported that signatures derived across many datasets can be more robust than those derived from one [19]. These studies differ from the present work in feature space and evaluation design, so their published areas under the curve and hazard ratios are not directly comparable with the C-indices reported here. The present design imposed technical measurability on every validation platform before outcome screening, hashed the panel file before external scoring and gave every molecular learner identical features. It also used paired bootstrap comparisons to test whether small point-estimate differences were credible, and allowed calibration and stability criteria to constrain the conclusion. These choices address documented sources of optimism in high-dimensional prognostic research [25,26,57], and the stage benchmark places the transported signal in its clinical context.

Several differences between cohorts are likely to have shaped these estimates. The platforms differ in probe content and dynamic range, from RNA sequencing to three Affymetrix designs, and 4 of the 12 genes were expressed near the lower end of at least one array. Case mix differed: GSE31210 contained only stage I–II tumors from a Japanese cohort with many never-smokers and *EGFR*-mutant tumors, whereas GSE72094 and GSE68465 included stage III disease. Event counts ranged from 35 to 236 and median follow-up from 2.4 to 6.3 years, which changed both the precision of each estimate and the horizons that could be evaluated. The time origin differed, with TCGA measured from diagnosis and the GEO cohorts from around the time of resection. Cohort-local standardization removes average differences in expression between cohorts and therefore any difference in average predicted risk; this helps ranking to transport but forces each cohort’s risk distribution to resemble that of TCGA. That the panel kept a consistent direction of association and an independent stage-adjusted effect across this heterogeneity supports its transportability. The non-proportional hazards in GSE68465, the cohort with the longest follow-up, suggest that the panel is most informative for earlier events.

SurvivalPFN replaces per-dataset weight learning with an inference procedure amortized across many prior survival tasks [30], in the same family as tabular foundation models that perform well on small data through learned in-context inference [29]. We use the term foundation model only in this technical sense. SurvivalPFN is described in a preprint that has not been peer-reviewed, and its advantage here was small. Such a prior may regularize prediction when event counts are limited, and SurvivalPFN gave the highest point estimate among the locked models in all three cohorts. The small differences from panel Cox, however, do not establish superiority or formal equivalence. These results show that amortized inference was competitive with regularized Cox under the tested specifications, without establishing that a more complex learner added meaningful discrimination. Shared-patient paired uncertainty was more informative here than overlapping marginal confidence intervals. With early stopping on a held-out validation split, DeepSurv reached C-indices of 0.638, 0.591 and 0.643, comparable to SurvivalPFN, which confirms that the panel supports similar discrimination across very different learners. The ordering of the tree-based methods varied by cohort, and post-lock XGBoost did not exceed SurvivalPFN or panel Cox. New models are accordingly best benchmarked against a well-regularized statistical model on identical inputs, with external cohorts withheld from every development decision [53,55].

Clinical use requires absolute risk at defined horizons rather than ranking alone. A patient with an estimated 20% rather than 40% five-year mortality may enter a different surveillance or trial pathway, and concordance cannot establish whether either estimate is accurate [24]. Both locked models reproduced average 3-year risk in the primary cohorts, which is encouraging for transport. Calibration slopes below 1 show that predicted risks were more spread out than observed outcomes, as is common when models are transported, and the overprediction in early-stage GSE31210 was shared by a stage-only model. TCGA-derived conformalization improved distributional calibration in GSE68465 but increased the integrated Brier score in GSE72094 and GSE31210. The corrections were learned on a held-out TCGA subset of 152 patients with 55 deaths, whereas the target cohorts differ in platform, era, stage composition, follow-up and censoring. Quantile correction also rests on exchangeability assumptions that biological and technical transport strain. Cohort-local standardization centers each cohort’s predictions on the TCGA risk distribution, which cannot be correct for a cohort restricted to early-stage disease. Differences in time origin and treatment era further alter the baseline hazard independently of the panel. GSE31210 contributed few deaths, which bounds tail assessment, and the implementation used a finite grid with Kaplan–Meier conditional sampling. Calibration is accordingly a property to be established at the horizon and in the population where the model will be applied, most likely after local recalibration of the baseline hazard.

The biological analyses support association rather than mechanism. Eleven of the 12 genes differed between tumor and the patient’s own normal lung, but a tumor-normal difference shows that a gene’s expression changes with malignancy, not why it predicts survival. No pathway enrichment or protein interaction network linked the genes, whether the whole genome or the 1232-gene selection universe was used as background. Experimental evidence links *TFAP2A* to LUAD glycolysis and progression [59], and its large paired increase and higher-risk association here are directionally consistent with that report, although the present observational data cannot test a causal mechanism. *NTSR1* has been reported to promote epithelial–mesenchymal transition and metastasis through Wnt/β-catenin signaling in LUAD [60]. *CPS1* expression has been associated with LUAD prognosis, and later experimental work connected CPS1-dependent ammonia handling to ROS/AMPK/P53/LKB1 signaling [61,62]. *CTNND2* fits the published picture less well. δ-catenin, which it encodes, has been reported to promote LUAD tumorigenesis and metastasis [63], yet *CTNND2* expression was lower in tumors than in matched normal lung, and higher expression was associated with longer survival in TCGA and GSE72094. The discrepancy may reflect differences between transcript and protein, between the cell populations sampled or between experimental systems, and it cautions against reading the panel genes as drivers. *KRT81* raises a different concern: it has been studied as a recurrence-associated locus in non-small-cell lung cancer and proposed as a marker of squamous cell carcinoma [64], so its signal could reflect mixed histology. Three observations argue against this in the present data: TCGA-CDR classified all 504 discovery patients as adenocarcinoma; excluding the 17 patients with non-adenocarcinoma labels in the flattened diagnosis field left *KRT81* associated with survival; and *KRT81* was associated with higher risk in both primary external cohorts, which contain only adenocarcinomas. Squamous differentiation within adenocarcinomas cannot, however, be excluded. Finally, selection frequency did not predict which genes would transport, because sign agreement was at least as common among the less stable genes. Stability as estimated here reflects competition among correlated candidates within TCGA rather than reliability across platforms.

The conditions under which these estimates apply follow directly from the design. All cohorts are retrospective and drawn from earlier treatment eras, so the estimates describe prognosis under historical management, and re-evaluation in contemporary perioperative cohorts is the natural next step [5,6]. No RNA-sequencing validation cohort was included in the locked design, so transport from RNA sequencing to RNA sequencing was not tested; GSE81089 and CPTAC-LUAD [65] would allow that comparison. GSE72094 and part of GSE68465 originate from the same institution; no overlap was detected, and excluding the shared site did not change the results, but overlap cannot be ruled out entirely without patient identifiers. Survival in TCGA is measured from diagnosis and in the GEO cohorts from around the time of resection, and stage in GSE68465 had to be reconstructed from pathological T and N categories. Several analyses, including the stage benchmark, were specified after the lock and should be read as hypothesis-generating, and gene-level directions are univariable associations rather than independent effects. Cohort-local median and median absolute deviation standardization requires a group of target patients and limits the transport of absolute risk, so a single-sample preprocessing rule is the necessary step towards individual-patient use. Overall survival reflects competing mortality and treatment as well as tumor biology, and symbol-level platform mapping leaves residual probe and isoform ambiguity that a purpose-built assay would resolve. SurvivalPFN has not yet been described in peer-reviewed form, and GSE31210, with 35 deaths, is underpowered.

These analyses also define the next steps. The full 12-gene panel preserved discrimination in early-stage disease where its six-gene core did not, and the panel added independent information to stage without exceeding it, so a combined stage-and-panel model is the natural candidate for prospective evaluation. A purpose-built assay could replace symbol-level mapping for genes measured near the detection limit of older arrays. Experimental work should confirm RNA and protein expression in independent LUAD tissue and perturb the strongest candidates, particularly *TFAP2A*, *CTNND2* and *KRT81*, in more than one model system. Clinical development requires the assay and intended use to be fixed before a new multi-center study. The relevant comparison is then incremental performance above a complete clinicopathological baseline that includes stage, performance status, smoking history, driver status and treatment, together with calibration at the decision-relevant horizon and, once a threshold and an action are defined, decision-curve analysis [58,66]. Because the panel measures a single molecular layer, integration with other omics layers could be explored, although multi-omics designs bring tradeoffs in cost, missing data and interpretability that have to be weighed against any gain in prediction [67]. If the intended purpose is adjuvant treatment selection, a predictive interaction with treatment rather than general prognosis must be demonstrated [8].

## 5. Conclusions

A 12-gene panel selected in TCGA and fixed for external evaluation transferred prognostic information across RNA sequencing and three microarray platforms. Its score remained associated with survival after adjustment for pathological stage, and panel-defined risk groups separated overall survival in every cohort. The panel did not, however, outperform pathological stage: stage alone discriminated at least as well as the panel alone in every cohort, and the higher discrimination of the combined stage-and-panel model in two of three cohorts was not statistically significant. Regularized Cox regression on the same genes performed as well as more complex learners, and average absolute risk was reproduced in both primary cohorts. These findings support prospective evaluation of the panel only as an adjunct to pathological stage, with calibration established in the target population. The locked validation framework used here can be applied to other candidate signatures.

## Figures and Tables

**Table 1:** Patient characteristics, prespecified analysis roles and endpoint information for the discovery and validation cohorts.

Characteristic	TCGA-LUAD	GSE72094	GSE68465	GSE31210
Role	Discovery	Primary external	Primary external	Early-stage stress test
Platform	RNA-seq (STAR counts)	GPL15048 array	GPL96 array	GPL570 array
Patients/deaths	504/182	398/113	442/236	226/35
Age, median (IQR), years	66 (59–73)	70 (64–76)	65 (58–72)	61 (55–65)
Male, n (%)	232 (46.0)	176 (44.2)	223 (50.5)	105 (46.5)
Smoking: ever/never/unknown	418/72/14	300/31/67	300/49/93	111/115/0
Stage I/II/III/IV/missing	270/120/81/25/8	254/67/57/15/5	276/95/68/0/3	168/58/0/0/0
Survival time origin	Diagnosis	Not stated (resected)	Not stated (resected)	Not stated (resected)
Median follow-up, years (95% CI)	2.4 (2.2–2.7)	2.5 (2.4–2.6)	6.3 (6.0–6.9)	5.1 (4.9–5.2)
3-year overall survival, %	62.6	63.3	67.6	90.9

IQR, interquartile range. Stage was taken from TCGA-CDR for TCGA-LUAD; GSE68465 stage was grouped from pathological T and N categories with M0 assumed, so no stage IV is recorded. Follow-up was estimated by the reverse Kaplan–Meier method, and 3-year survival by the Kaplan–Meier method.

**Table 2:** Locked external discrimination for all six models in the three validation cohorts. Confidence intervals are 95% patient-level bootstrap intervals from 1000 resamples. Models are listed in descending order of C-index within each cohort.

Cohort	Model	C-Index (95% CI)	n/Events
GSE72094	SurvivalPFN	0.631 (0.572–0.689)	398/113
GSE72094	Panel Cox	0.620 (0.563–0.680)	398/113
GSE72094	Random survival forest	0.612 (0.552–0.674)	398/113
GSE72094	Gradient boosting survival	0.594 (0.534–0.652)	398/113
GSE72094	DeepSurv	0.569 (0.507–0.629)	398/113
GSE72094	Age/sex Cox	0.544 (0.489–0.601)	398/113
GSE68465	SurvivalPFN	0.583 (0.541–0.624)	442/236
GSE68465	Age/sex Cox	0.578 (0.535–0.620)	442/236
GSE68465	Panel Cox	0.578 (0.534–0.618)	442/236
GSE68465	Random survival forest	0.570 (0.527–0.608)	442/236
GSE68465	Gradient boosting survival	0.558 (0.515–0.594)	442/236
GSE68465	DeepSurv	0.540 (0.501–0.582)	442/236
GSE31210	SurvivalPFN	0.665 (0.581–0.750)	226/35
GSE31210	Gradient boosting survival	0.661 (0.574–0.743)	226/35
GSE31210	Panel Cox	0.659 (0.571–0.751)	226/35
GSE31210	DeepSurv	0.624 (0.532–0.714)	226/35
GSE31210	Random survival forest	0.621 (0.524–0.717)	226/35
GSE31210	Age/sex Cox	0.597 (0.485–0.704)	226/35

**Table 3:** Paired differences in Harrell’s C-index between models in each external cohort. Each value is the first model minus the second with a 95% interval from 2000 paired bootstrap resamples; an asterisk marks an interval that excludes zero. Comparisons involving stage are restricted to patients with recorded stage.

Comparison	GSE72094	GSE68465	GSE31210
SurvivalPFN − age/sex Cox	0.086 (0.009 to 0.166) *	0.005 (−0.053 to 0.062)	0.068 (−0.038 to 0.179)
SurvivalPFN − panel Cox	0.011 (−0.014 to 0.036)	0.005 (−0.009 to 0.020)	0.006 (−0.026 to 0.039)
Panel Cox − stage-only Cox	−0.019 (−0.093 to 0.058)	−0.083 (−0.133 to −0.034) *	−0.018 (−0.141 to 0.100)
SurvivalPFN − stage-only Cox	−0.008 (−0.088 to 0.072)	−0.076 (−0.126 to −0.027) *	−0.013 (−0.130 to 0.103)
Stage + panel − stage-only Cox	0.029 (−0.024 to 0.081)	−0.008 (−0.043 to 0.025)	0.040 (−0.062 to 0.135)
Stage + age/sex + panel − stage + age/sex Cox	0.027 (−0.026 to 0.080)	−0.020 (−0.055 to 0.015)	0.018 (−0.096 to 0.126)
Six-gene − 12-gene panel Cox	0.001 (−0.036 to 0.039)	−0.005 (−0.038 to 0.028)	−0.104 (−0.179 to −0.036) *
Six-gene − 12-gene SurvivalPFN	−0.001 (−0.038 to 0.037)	−0.001 (−0.034 to 0.032)	−0.115 (−0.184 to −0.051) *

**Table 4:** Discrimination of stage-based, panel-based and combined models in patients with recorded stage. Models were fitted in TCGA (496 patients with TCGA-CDR stage) and scored unchanged; values are Harrell’s C-index with 95% bootstrap intervals. The last two rows give the hazard ratio (HR) per standard deviation (SD) of the locked panel score adjusted within each cohort, with the likelihood-ratio *p* value.

Model	GSE72094 (*n* = 393; 111 Deaths)	GSE68465 (*n* = 439; 235 Deaths)	GSE31210 (*n* = 226; 35 Deaths)
Stage-only Cox	0.634 (0.582–0.682)	0.661 (0.627–0.693)	0.678 (0.594–0.752)
Stage + age/sex Cox	0.630 (0.572–0.683)	0.687 (0.648–0.723)	0.685 (0.577–0.785)
Panel Cox	0.616 (0.553–0.673)	0.578 (0.535–0.619)	0.659 (0.567–0.750)
SurvivalPFN	0.627 (0.565–0.685)	0.584 (0.541–0.626)	0.665 (0.574–0.753)
Stage + panel Cox	0.663 (0.608–0.717)	0.653 (0.613–0.689)	0.718 (0.635–0.797)
Stage + age/sex + panel Cox	0.657 (0.599–0.713)	0.667 (0.627–0.703)	0.703 (0.609–0.789)
Panel score HR per SD, stage-adjusted	1.36 (1.12–1.65); *p* = 0.002	1.12 (0.98–1.28); *p* = 0.087	1.56 (1.11–2.17); *p* = 0.009
Panel score HR per SD, adjusted for stage, age and sex	1.38 (1.13–1.68); *p* = 0.002	1.11 (0.97–1.27); *p* = 0.121	1.51 (1.07–2.14); *p* = 0.019

**Table 5:** Post-selection biological evaluation in 58 matched tumor-normal pairs. Paired *d*_z_ is tumor minus solid-tissue normal; *p* values are from two-sided Wilcoxon signed-rank tests and false discovery rates are Benjamini–Hochberg corrected across the 12-gene panel. Stage rho and stage FDR use TCGA-CDR stage (*n* = 496).

Gene	Paired dz	Tumor-Normal FDR	Stage Rho	Stage FDR	External Direction Matches	Stability
*FAIM2*	−0.23	0.0129	−0.17	0.000928	2/3	0.785
*NTSR1*	0.41	0.00707	0.05	0.276	2/3	0.725
*CPS1*	0.45	0.00707	0.09	0.0815	3/3	0.710
*KRT81*	0.84	8.25 × 10^−9^	0.07	0.173	3/3	0.630
*IGFBP1*	0.37	0.0292	0.18	0.000547	3/3	0.625
*FLNC*	−0.27	0.024	0.14	0.00597	2/3	0.600
*CIDEC*	0.14	0.967	0.15	0.00253	1/3	0.560
*FBN2*	0.56	0.00012	0.08	0.116	2/3	0.540
*CTNND2*	−0.97	1.75 × 10^−6^	−0.12	0.0202	3/3	0.540
*CCL20*	0.41	0.00707	0.08	0.0969	3/3	0.515
*TFAP2A*	1.60	8.27 × 10^−10^	0.08	0.0969	3/3	0.485
*MAGEA8*	0.30	0.0292	−0.01	0.89	3/3	0.420

## Data Availability

TCGA-LUAD data are available from the NCI Genomic Data Commons (https://portal.gdc.cancer.gov/, accessed on 3 August 2026). GSE72094, GSE68465 and GSE31210 are available from NCBI GEO (https://www.ncbi.nlm.nih.gov/geo/, accessed on 3 August 2026). Analysis code is available at https://github.com/falrumaihi/luad-survival-benchmark, accessed on 3 August 2026. The curated TCGA-CDR clinical table is available from the NCI Genomic Data Commons PanCanAtlas publication page (https://gdc.cancer.gov/about-data/publications/pancanatlas, accessed on 3 August 2026). Scripts for the post-lock analyses are included in the same repository.

## Supplementary Materials

The following supporting information can be downloaded at: https://www.mdpi.com/article/10.3390/genes17101264/s1, Text S1: Supplementary Methods; Figure S1: Kaplan–Meier overall survival for (a) TCGA-LUAD, (b) GSE72094, (c) GSE68465 and (d) GSE31210. Follow-up and censoring support differ markedly across the four cohorts, which constrains which time horizons can be estimated in each; Figure S2: Preprocessing audit. (a) Outcome-blind feature reduction from the raw input matrix through count filtering and the protein-coding restriction to the 2000 model features. (b) Number of panel-eligible gene symbols mapped on each external platform. (c) Patient and death counts contributed by each external cohort; Figure S3: Selection behavior and internal resampling. (a) Bootstrap selection frequency against median absolute elastic-net coefficient for each panel gene; red points met the prespecified 0.60 threshold. (b) Descriptive post-selection internal fold C-index by model. These internal estimates followed panel selection on the full discovery cohort and are not selection-unbiased; Figure S4: Univariable Cox coefficients in TCGA plotted against the corresponding external coefficient for (a) GSE72094, (b) GSE68465 and (c) GSE31210. Red points share the TCGA sign and blue points reverse it. Direction agreement was 75%, 83% and 92% respectively; Figure S5: Overall survival by TCGA-derived tertile of the panel Cox score in (a) GSE72094, (b) GSE68465 and (c) GSE31210. Cut-points were fixed in TCGA before scoring; hazard ratios compare the high-risk with the low-risk group; Figure S6: Calibration of predicted 3-year risk by quintile of predicted risk for panel Cox, raw SurvivalPFN and conformalized SurvivalPFN in (a) GSE72094, (b) GSE68465 and (c) GSE31210. Observed risk is one minus the Kaplan–Meier estimate, with 95% confidence intervals; the dashed line marks perfect calibration; Figure S7: D-calibration histograms for raw SurvivalPFN predictions in (a) GSE72094, (b) GSE68465 and (c) GSE31210, and for the conformalized predictions in (d), (e) and (f). The dashed line marks the expected count under perfect calibration. GSE72094 was already near-uniform before correction, GSE68465 showed the U-shaped excess that conformalization flattened, and GSE31210 declined monotonically both before and after correction; Figure S8: Extended discrimination by metric and time horizon in (a) GSE72094, (b) GSE68465 and (c) GSE31210. Grey cells labeled NE indicate values that were not estimable because censoring or event support was insufficient. No missing performance value was imputed; Figure S9: Evidence synthesis and the interpretation boundary. (a) Outcome of the five substantive criteria and the procedural sixth step. (b) The evidence grade that follows from applying them conjunctively; Figure S10: External C-index with 95% bootstrap confidence intervals for the three post-lock sensitivity models in each cohort. These models were specified after the primary lock and cannot affect the prespecified interpretation criteria; Figure S11: Median expression of each panel gene as a percentile of all genes measured on the same platform. Red outlines mark microarray values below the 10th percentile, which identified *IGFBP1*, *KRT81*, *MAGEA8* and *NTSR1* for the expression-level sensitivity analysis; Figure S12: External C-index of 1000 random 12-gene panels drawn from the 1232-gene selection universe and fitted with the panel Cox specification in TCGA, in (a) GSE72094, (b) GSE68465 and (c) GSE31210. The red line marks the locked panel and the dashed line the median of the random panels; Table S1: Event support by prediction horizon in each external cohort. Deaths before the horizon, patients still under observation beyond it, patients censored before it, the truncation time used for Uno’s C-index and whether the cumulative/dynamic AUC could be estimated; Table S2: Locked panel stability and univariable direction transport. External direction matches count agreement with the TCGA coefficient sign across GSE72094, GSE68465 and GSE31210; Table S3: Selection frequencies with the univariable screen repeated inside each of 200 bootstrap replicates (fixed penalty 0.0597), shown for the 20 most frequently selected genes, alongside the frequencies from the locked selection; Table S4: Univariable Cox coefficients, direction labels and TCGA-agreement flags for all 12 panel genes in TCGA-LUAD and each external cohort (48 gene-cohort rows); Table S5: Univariable Cox hazard ratios per robust standard-deviation unit, with 95% confidence intervals, for every panel gene in every cohort. FDR is Benjamini–Hochberg corrected within each cohort; Table S6: Descriptive internal cross-validation results, giving the per-fold C-index, test-set size and event count for every model. CPU models contributed three repeats of five folds; GPU models contributed one repeat of five folds; Table S7: Overall survival by tertile of the panel Cox score, with cut-points fixed in TCGA. Survival is the Kaplan–Meier estimate in percent with 95% confidence interval; the last row for each cohort gives the Cox hazard ratio for the high- versus low-risk group; Table S8: Paired bootstrap comparisons of SurvivalPFN against the age and sex Cox model and against panel Cox in each cohort, with the delta C-index, its 95% confidence interval and the number of paired replicates; Table S9: Association of the standardized panel Cox and SurvivalPFN scores with survival within each external cohort, adjusted for categorical stage, and for stage, age and sex. HR is per standard deviation; *p* values are from likelihood-ratio tests of the added score. The last rows give panel Cox discrimination among stage I patients; Table S10: Calibration of transported models. IBS is the integrated Brier score with a 1000-replicate bootstrap interval where shown; the reference is a covariate-free Kaplan–Meier estimate in the target cohort; IPA is the index of prediction accuracy (1 − IBS/reference IBS); the calibration slope is the coefficient of the transported prognostic index (3-year complementary log-log risk for SurvivalPFN) in the target cohort; O/E is observed over mean predicted 3-year risk; Table S11: Calibration metrics for raw and conformalized SurvivalPFN predictions in each cohort. The 10-bin D-calibration histogram behind each chi-squared statistic is plotted in full as Figure S7 and retained in the accompanying CSV; Table S12: Extended discrimination metrics for every model and cohort, giving Uno’s C-index, the truncation time in days and cumulative/dynamic area under the curve at 1, 3 and 5 years. Empty cells were not estimable given the available censoring and event support; Table S13: Paired tumor-normal results for all 12 genes in the 58 matched pairs, giving the median tumor and normal values on the variance-stabilized scale, the median paired difference, paired Cohen’s dz, the Wilcoxon signed-rank statistic, the raw *p* value and the Benjamini–Hochberg false discovery rate. Direction is shown only for genes with a false discovery rate below 0.05; *CIDEC* did not differ and is shown as unchanged; Table S14: Stage trend results for all 12 genes across the 496 patients with TCGA-CDR pathological stage, giving Spearman’s rho, the raw *p* value and the false discovery rate; Table S15: Functional enrichment output from g:Profiler across Gene Ontology, Reactome, KEGG, WikiPathways and CORUM. The query returned an empty result set, which is reported here rather than omitted. This query used the whole genome as background; the analysis against the 1232-gene selection universe is given in Table S17; Table S16: STRING v12 network output at medium confidence 0.4. The edge list returned empty, which is reported here rather than omitted; Table S17: Functional enrichment of the 12 panel genes against the 1232-gene selection universe as background. For each source the number of terms tested, the number with adjusted *p* < 0.05, the smallest adjusted *p* value and the top-ranked term are shown; Table S18: Outcome of the five substantive prespecified interpretation criteria and the procedural sixth step. Three of the five substantive criteria were met; Table S19: External discrimination for the three post-lock sensitivity models in each cohort, with 95% bootstrap confidence intervals. Every row in this table was specified after the primary model lock; Table S20: Sensitivity analyses specified after the lock. C-index values are Harrell’s C with 95% bootstrap intervals; differences are paired against the corresponding locked 12-gene model. The last rows summarize the GSE72094/GSE68465 overlap checks, in which sites other than Moffitt cannot overlap with GSE72094 and act as the reference; Table S21: External C-index of the locked panel against 1000 random 12-gene panels drawn from the 1232-gene selection universe, each fitted with the panel Cox specification in TCGA. The empirical *p* value is the proportion of random panels with a C-index at least as high as the locked panel; Table S22: Proportional hazards tests based on scaled Schoenfeld residuals against rank-transformed time. The global statistic for the 12-gene TCGA model is the sum of the 12 one-degree-of-freedom statistics.
