# Supplementary material for: Cross-Platform External Validation of a Locked 12-Gene Prognostic Panel in Lung Adenocarcinoma Benchmarked Against Pathological Stage
Source: Genes (Basel). 2026 Oct 11;17(10):1264. doi: 10.3390/genes17101264 (PMC13704482; doi:10.3390/genes17101264)
Supplement: Supplementary file 1 [file genes-17-01264-s001.zip › genes-4598855-supplementary.pdf]

Supporting information
Cross-Platform External Validation of a Locked 12-Gene Prognostic Panel in Lung Adenocarcinoma Benchmarked Against Pathological Stage

Faris Alrumaihi[1,*]

[1]Department of Medical Laboratories, College of Applied Medical Sciences, Qassim University, Buraydah 51452, Saudi Arabia

*Correspondence: f_alruaihi@qu.edu.sam

Text S1. Supplementary Methods

Raw-input preservation

The four root TCGA files are treated as immutable inputs. Every generated file is written under *artifacts/*, *external_data/*, *external_models/* or *manuscript/*, so that no analysis step can alter a source file. SHA-256 checksums for all inputs and outputs are recorded in the artifact manifest.

Patient-level construction

Barcodes were reduced to patient identifiers. Where several primary-tumor aliquots existed for one patient, the preferred vial was chosen first and raw library size resolved any remaining tie. Exclusion and aliquot-selection tables were retained in full. Survival time had to be present and strictly positive for a patient to enter the discovery cohort.

RNA-seq processing

GENCODE v36 identifiers were matched exactly, including *_PAR_Y* entries. Filtering required at least 10 counts in at least 51 of the 504 eligible patients. The DESeq2 variance-stabilizing transformation used *blind=TRUE* and *design=~1*. Protein-coding symbols were deduplicated by variance and the 2,000 most variable genes were selected without reference to outcomes.

Cross-platform harmonization

The discovery universe was restricted to genes measurable in TCGA and in all three GEO cohorts. External expression was never pooled with TCGA data. Each cohort was standardized independently using its own median and median absolute deviation (scaled by 1.4826, with the

standard deviation used when the median absolute deviation was zero), without using survival outcomes. This is a cohort-level normalization rule; single-patient deployment was not evaluated. Probes annotated to more than one gene symbol were discarded and the remaining probes for each symbol were collapsed to their per-sample median. Values were log2(x + 1) transformed when the 99th percentile of a downloaded matrix exceeded 100, which applied to GSE68465 and GSE31210; GSE72094 was supplied on a log scale.

Selection and locking

The univariable survival screen reduced the 1,232 shared genes to 250 candidates. Elastic-net Cox regression used *l1_ratio=0.9*, with alpha chosen by the one-standard-error rule. The screen was run once in the full discovery cohort; each bootstrap replicate refitted only the elastic net on the 250 screened genes at the fixed penalty. The panel size of 12 was set before selection, and the 0.60 frequency was a reporting standard rather than an inclusion rule. Two hundred bootstrap replicates ran across 12 workers. The resulting 12-gene file was hashed, and the hash, panel, seeds, model list and cohort roles were written to *artifacts/lock/model_spec.json* before any external scoring took place.

Validation and calibration

External metrics were computed on unchanged cohort outcomes. Confidence intervals used patient-level bootstrap resampling. Paired deltas reused the same resampled patients for both compared models. Conformalized survival distributions were derived only from a held-out TCGA calibration split; no external outcome contributed to the corrections. The 30% calibration subset contained 152 patients and 55 deaths. Panel Cox was also evaluated for calibration using the Breslow baseline hazard from TCGA, and both models were compared with a covariate-free Kaplan-Meier reference, the index of prediction accuracy, the calibration slope and the observed-to-predicted 3-year risk ratio.

Computing environment

Windows R 4.6.0 performed the DESeq2 preprocessing. Python 3.11.15 performed all remaining stages. DeepSurv early stopping in the locked run monitored the training loss. SurvivalPFN, DeepSurv and post-lock XGBoost Cox used CUDA on an NVIDIA RTX 4090 GPU. Feature-selection bootstraps used 12 CPU workers. The post-lock analyses ran under Python 3.12 with

scikit-survival 0.28.0, scikit-learn 1.9.1, lifelines 0.30.3, NumPy 2.5.3, pandas 2.3.3, SciPy 1.18.1 and PyTorch 2.14.1 on CPU with the SurvivalPFN v0.1 checkpoint. Refitting panel Cox and SurvivalPFN in this environment reproduced the locked external C-indices exactly.

Post-lock analyses

All analyses in this section were performed after the lock and did not change the panel, models or criteria. Pathological stage for TCGA was taken from the TCGA Pan-Cancer Clinical Data Resource (496 of 504 patients). GSE68465 stage was grouped from pathological T and N categories under the sixth edition of the AJCC system with M0 assumed: pT1-2N0 as stage I, pT1-2N1 or pT3N0 as stage II and other combinations as stage III; 3 patients with pNX or incomplete codes were left unstaged. Stage-only, panel and stage-plus-panel Cox models (L2 penalty 0.1, ordinal stage) were fitted in TCGA and scored unchanged. Within each external cohort, likelihood-ratio tests compared Cox models with categorical stage, with or without age and sex, before and after adding the standardized locked score. Random panels were 1,000 draws of 12 genes from the 1,232-gene universe, each fitted with the panel Cox specification. Proportional hazards were tested with scaled Schoenfeld residuals against rank-transformed time. Follow-up used the reverse Kaplan-Meier method. Expression level was the median expression of each gene ranked among all genes on the same platform. For cohort overlap, each GSE68465 patient was compared with every GSE72094 patient for identical clinical records (sex, age within 1 year, stage group, vital status and survival within 31 days) and for expression similarity across the 1,232 shared genes, standardized against that patient's correlations with all GSE72094 samples; the three GSE68465 sites outside Moffitt served as the null reference. DeepSurv was refitted with early stopping on a stratified 20% validation split (403 training and 101 validation patients, 36 validation deaths).

Supporting figures

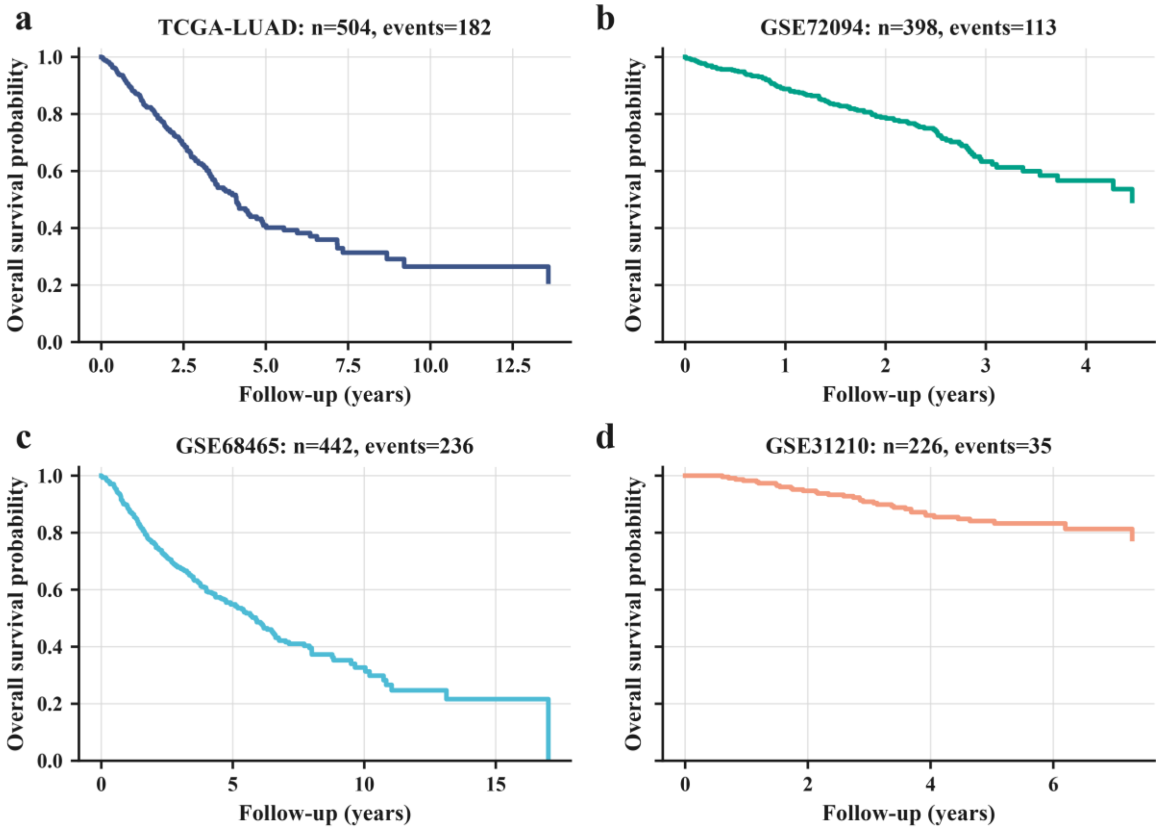

Figure S1. Kaplan-Meier overall survival for (a) TCGA-LUAD, (b) GSE72094, (c) GSE68465 and (d) GSE31210. Follow-up and censoring support differ markedly across the four cohorts, which constrains which time horizons can be estimated in each.

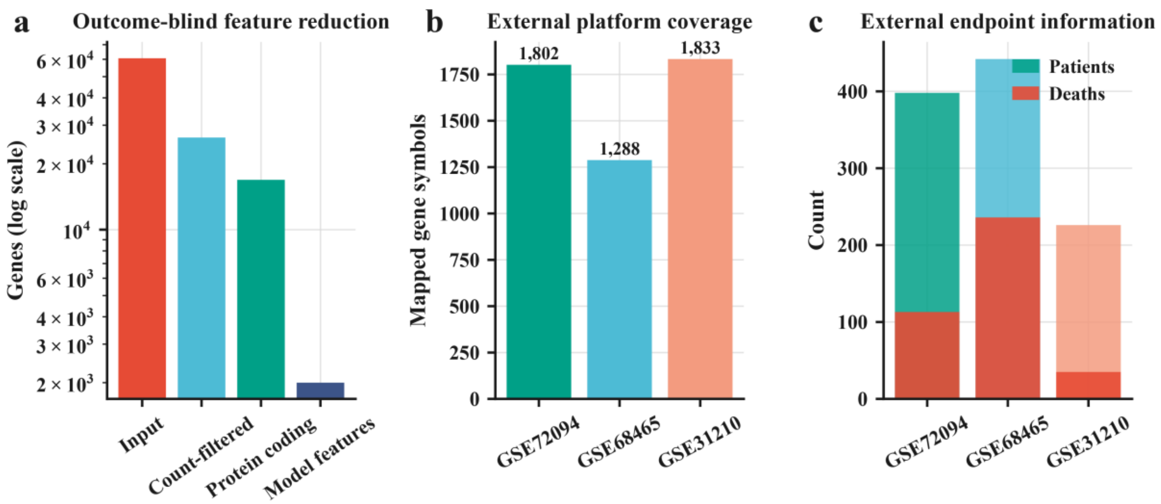

Figure S2. Preprocessing audit. (a) Outcome-blind feature reduction from the raw input matrix through count filtering and the protein-coding restriction to the 2,000 model features. (b) Number of panel-eligible gene symbols mapped on each external platform. (c) Patient and death counts contributed by each external cohort.

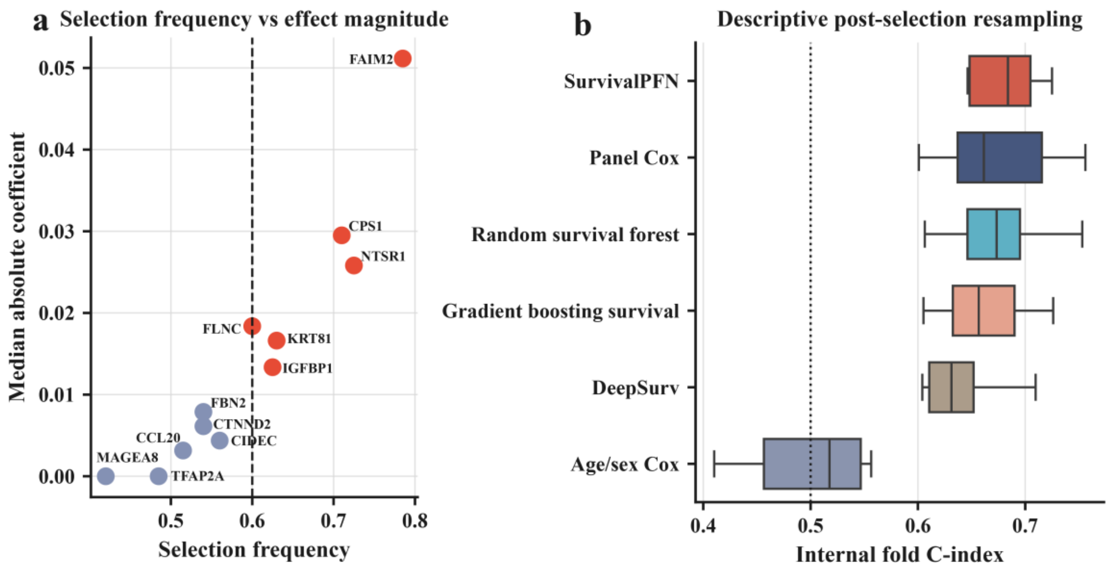

Figure S3. Selection behavior and internal resampling. (a) Bootstrap selection frequency against median absolute elastic-net coefficient for each panel gene; red points met the prespecified 0.60 threshold. (b) Descriptive post-selection internal fold C-index by model. These internal estimates followed panel selection on the full discovery cohort and are not selection-unbiased.

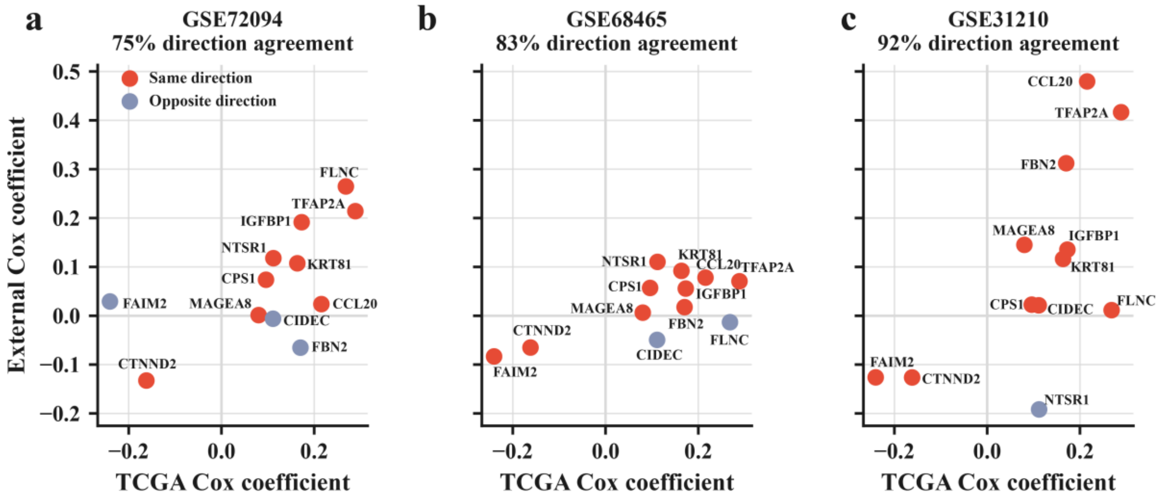

Figure S4. Univariable Cox coefficients in TCGA plotted against the corresponding external coefficient for (a) GSE72094, (b) GSE68465 and (c) GSE31210. Red points share the TCGA sign and blue points reverse it. Direction agreement was 75%, 83% and 92% respectively.

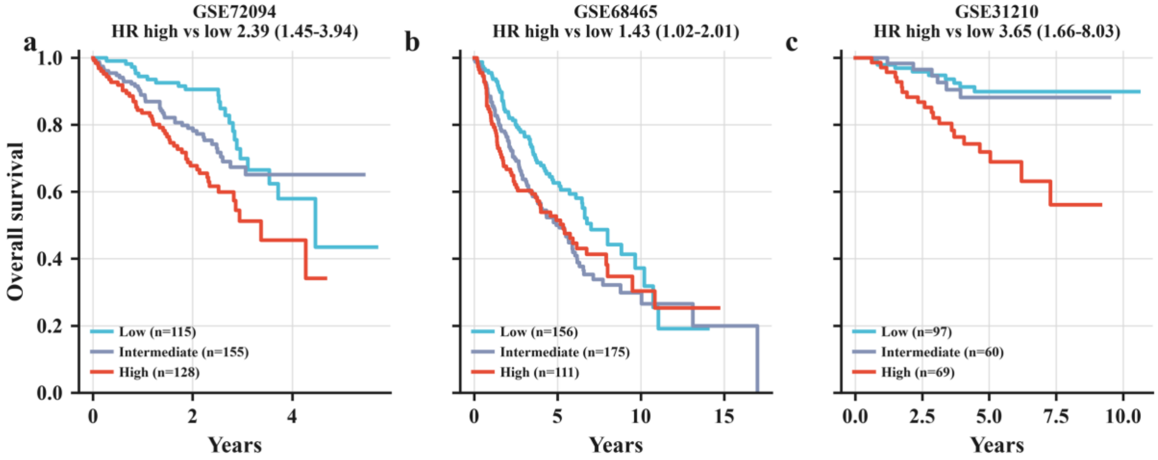

Figure S5. Overall survival by TCGA-derived tertile of the panel Cox score in (a) GSE72094, (b) GSE68465 and (c) GSE31210. Cut-points were fixed in TCGA before scoring; hazard ratios compare the high-risk with the low-risk group.

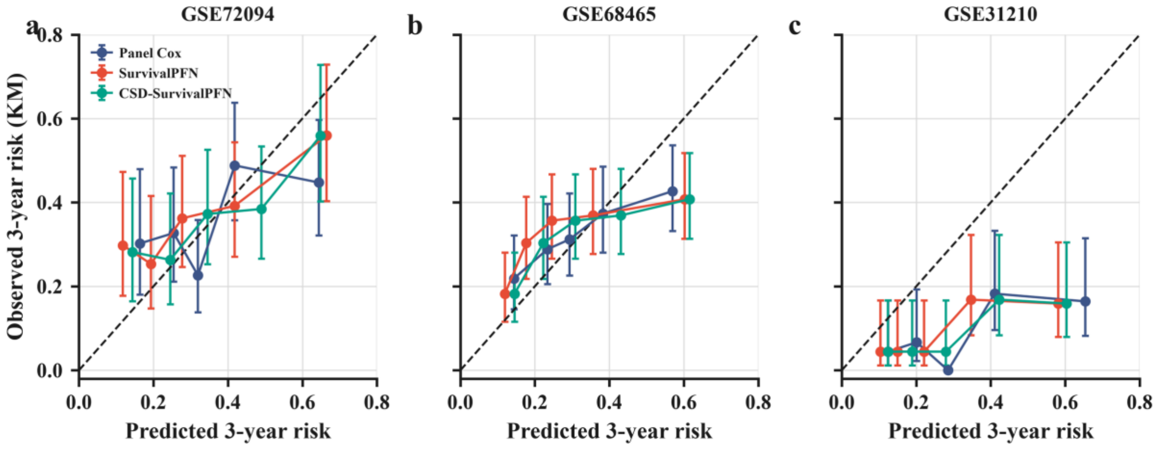

Figure S6. Calibration of predicted 3-year risk by quintile of predicted risk for panel Cox, raw SurvivalPFN and conformalized SurvivalPFN in (a) GSE72094, (b) GSE68465 and (c) GSE31210. Observed risk is one minus the Kaplan-Meier estimate, with 95% confidence intervals; the dashed line marks perfect calibration.

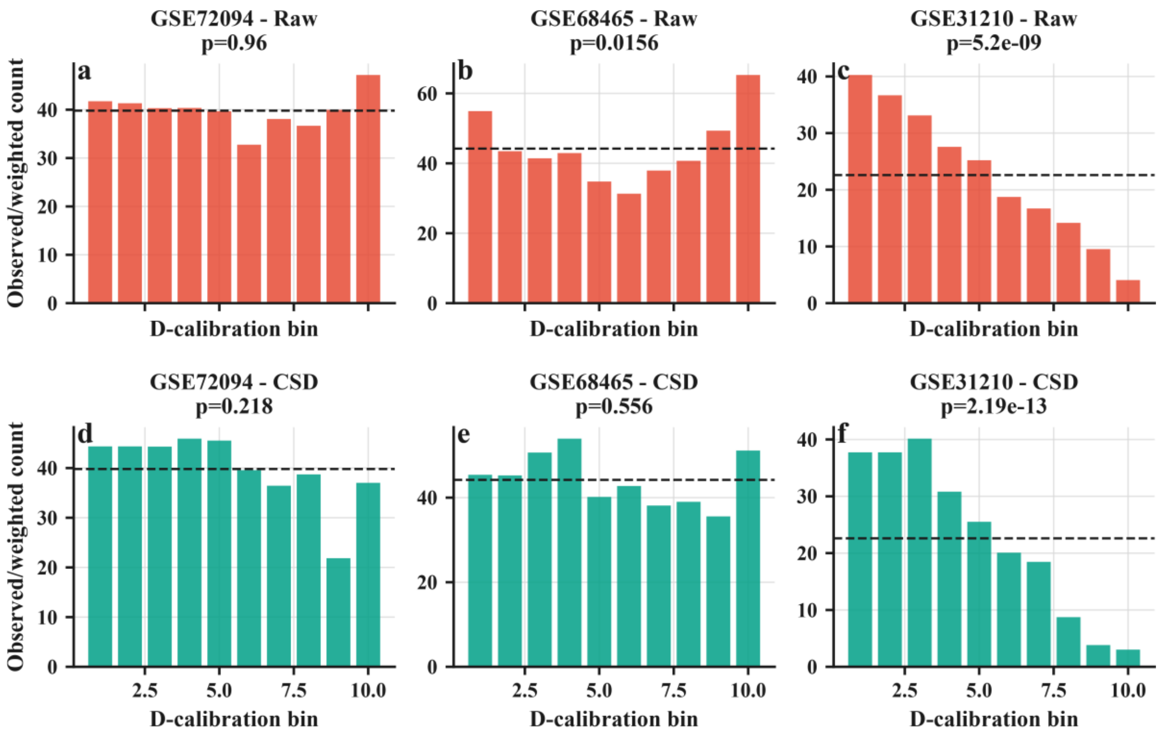

Figure S7. D-calibration histograms for raw SurvivalPFN predictions in (a) GSE72094, (b) GSE68465 and (c) GSE31210, and for the conformalized predictions in (d), (e) and (f). The dashed line marks the expected count under perfect calibration. GSE72094 was already near-uniform before correction, GSE68465 showed the U-shaped excess that conformalization flattened, and GSE31210 declined monotonically both before and after correction.

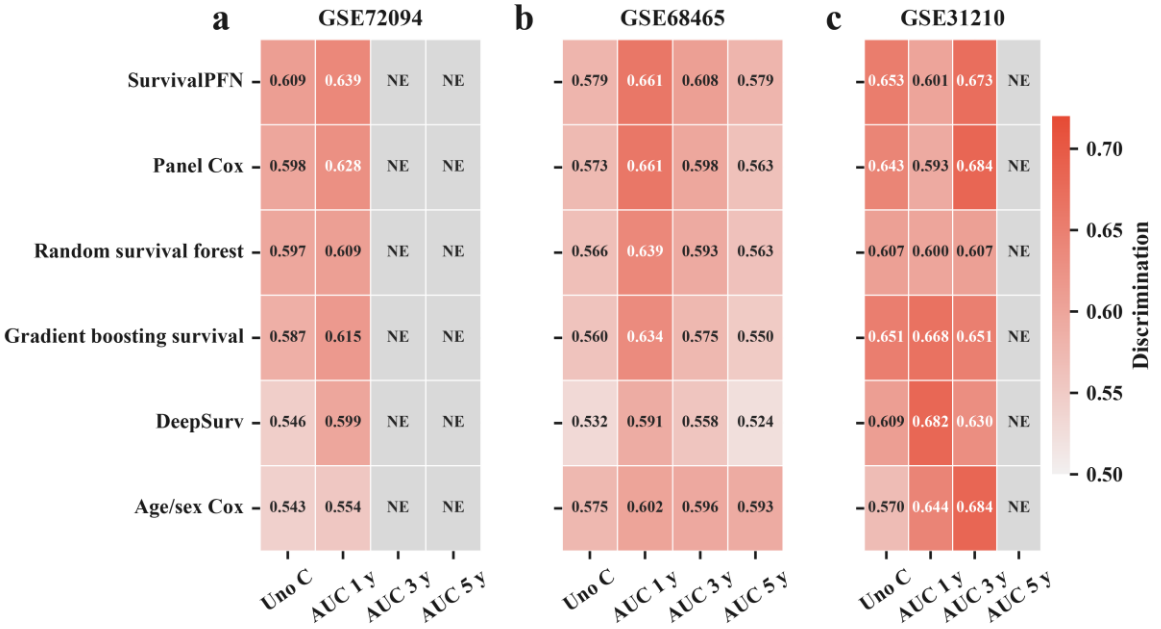

Figure S8. Extended discrimination by metric and time horizon in (a) GSE72094, (b) GSE68465 and (c) GSE31210. Grey cells labeled NE indicate values that were not estimable because censoring or event support was insufficient. No missing performance value was imputed.

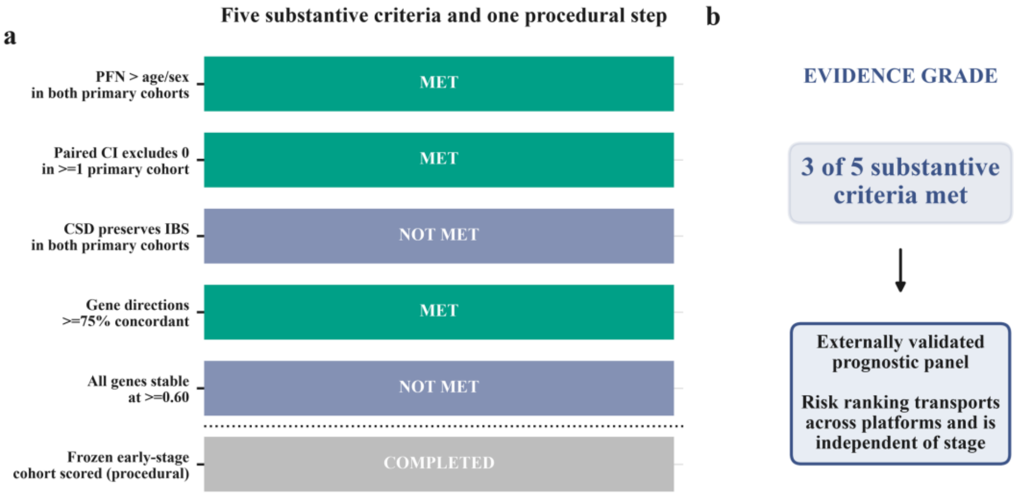

Figure S9. Evidence synthesis and the interpretation boundary. (a) Outcome of the five substantive criteria and the procedural sixth step. (b) The evidence grade that follows from applying them conjunctively.

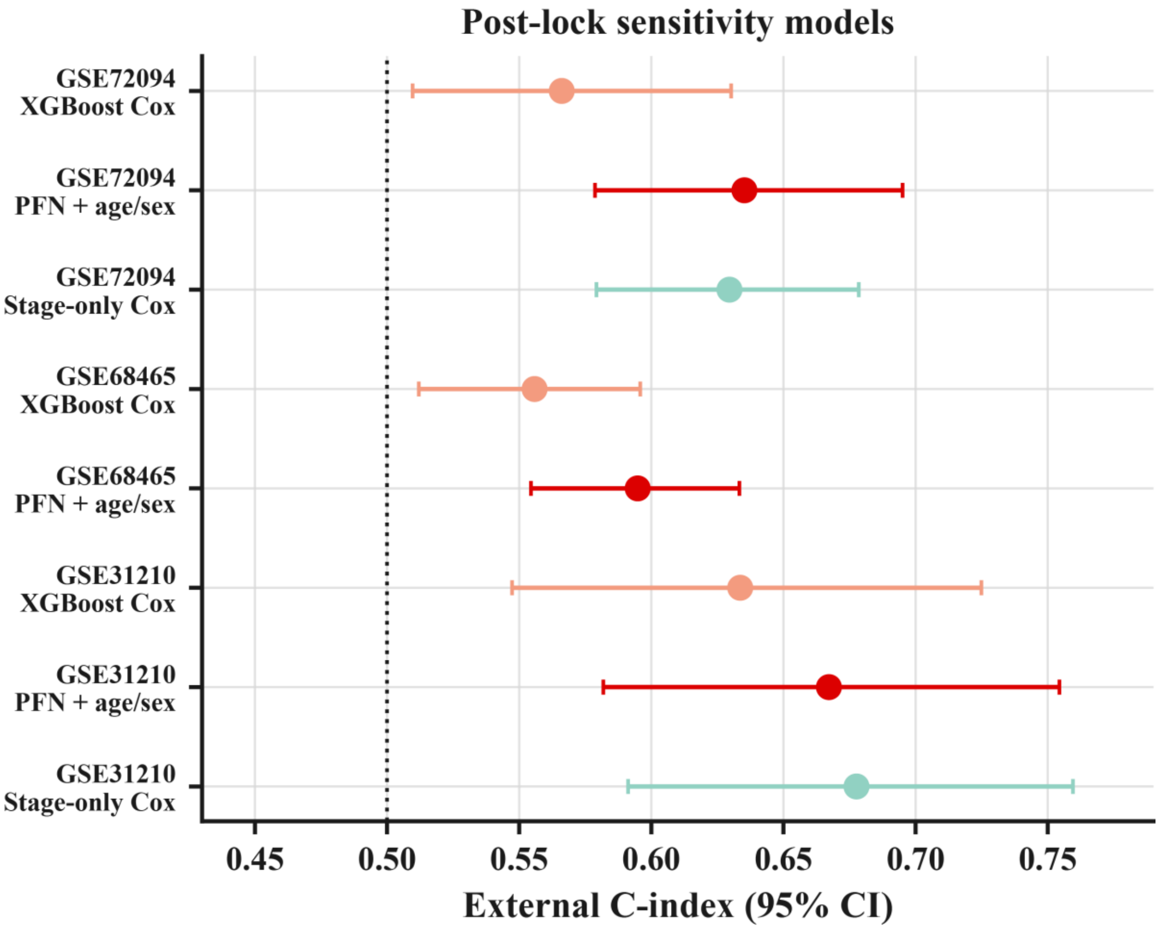

Figure S10. External C-index with 95% bootstrap confidence intervals for the three post-lock sensitivity models in each cohort. These models were specified after the primary lock and cannot affect the prespecified interpretation criteria.

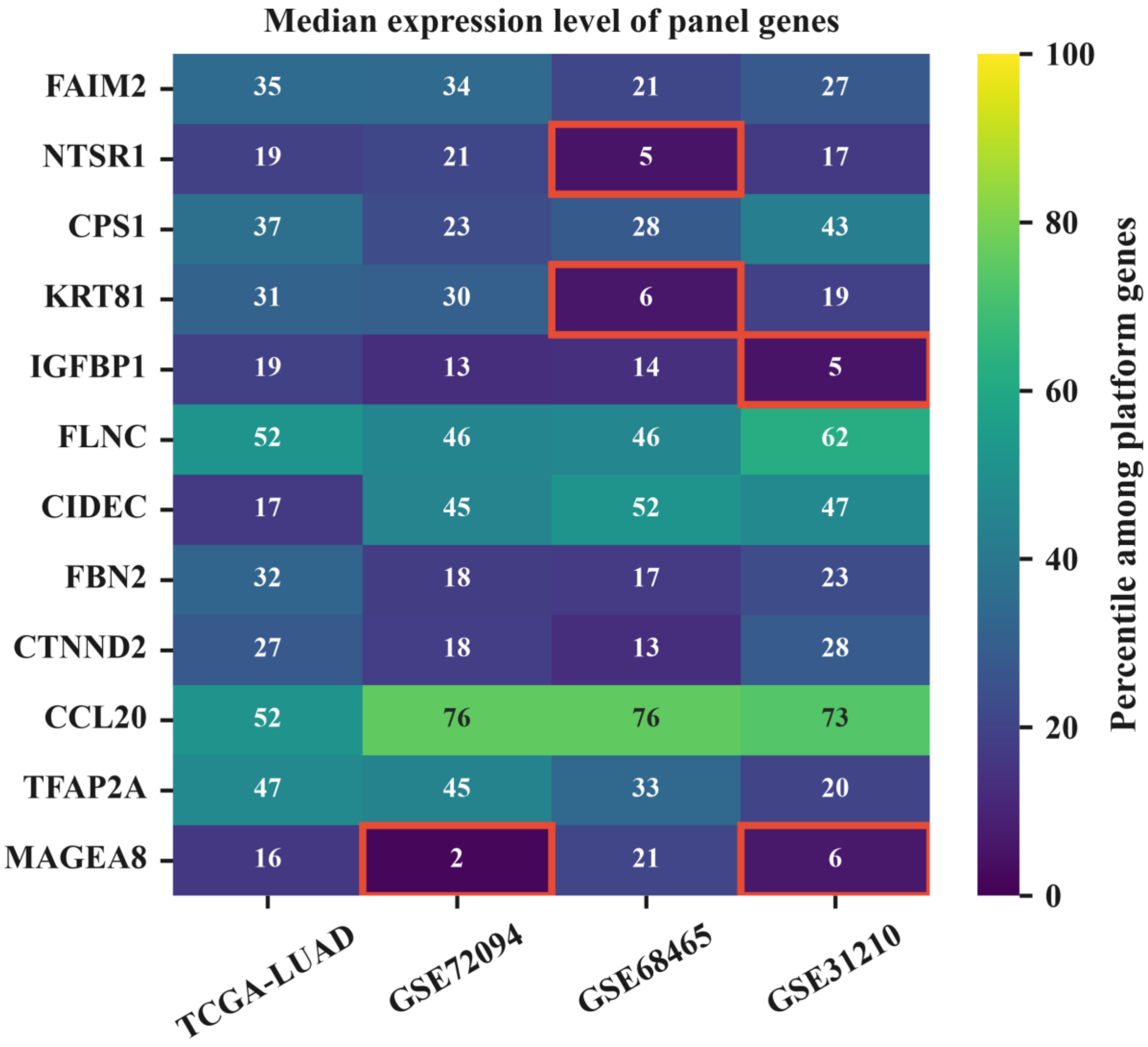

Figure S11. Median expression of each panel gene as a percentile of all genes measured on the same platform. Red outlines mark microarray values below the 10th percentile, which identified IGFBP1, KRT81, MAGEA8 and NTSR1 for the expression-level sensitivity analysis.

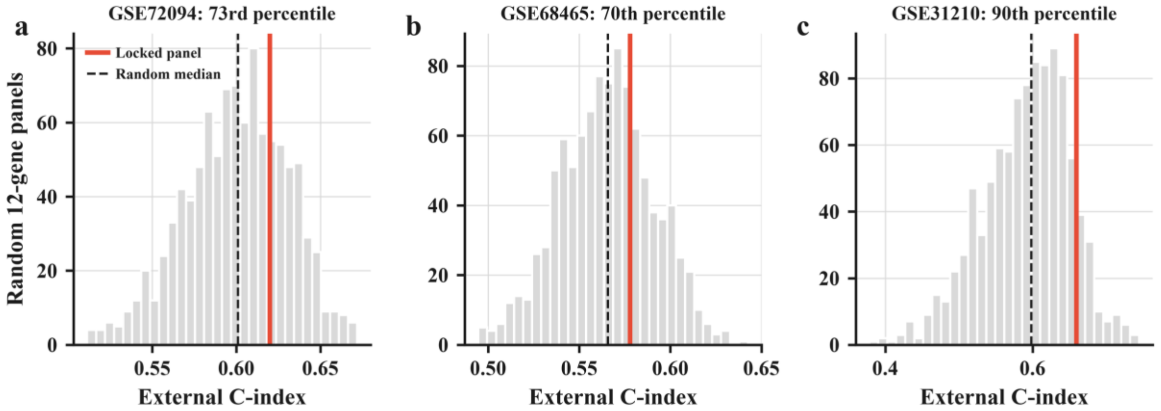

Figure S12. External C-index of 1,000 random 12-gene panels drawn from the 1,232-gene selection universe and fitted with the panel Cox specification in TCGA, in (a) GSE72094, (b) GSE68465 and (c) GSE31210. The red line marks the locked panel and the dashed line the median of the random panels.

Supporting tables

Table S1. Event support by prediction horizon in each external cohort. Deaths before the horizon, patients still under observation beyond it, patients censored before it, the truncation time used for Uno's C-index and whether the cumulative/dynamic AUC could be estimated.

Cohort	Horizon	Deaths before	At risk beyond	Censored before	Uno τ (days)	AUC estimable
GSE72094	1 year	43	332	23	1036	Yes
GSE72094	3 years	106	68	224	1036	No
GSE72094	5 years	113	5	280	1036	No
GSE68465	1 year	48	392	2	2382	Yes
GSE68465	3 years	140	278	24	2382	Yes
GSE68465	5 years	187	171	84	2382	Yes
GSE31210	1 year	4	222	0	1667	Yes
GSE31210	3 years	20	185	21	1667	Yes
GSE31210	5 years	32	102	92	1667	No

Table S2. Locked panel stability and univariable direction transport. External direction matches count agreement with the TCGA coefficient sign across GSE72094, GSE68465 and GSE31210.

Rank	Gene	Selection frequency	TCGA direction	External direction matches	Passes 0.60
1	FAIM2	0.785	lower risk	2/3	Yes
2	NTSR1	0.725	higher risk	2/3	Yes
3	CPS1	0.710	higher risk	3/3	Yes
4	KRT81	0.630	higher risk	3/3	Yes
5	IGFBP1	0.625	higher risk	3/3	Yes
6	FLNC	0.600	higher risk	2/3	Yes
7	CIDEC	0.560	higher risk	1/3	No
8	FBN2	0.540	higher risk	2/3	No
9	CTNND2	0.540	lower risk	3/3	No
10	CCL20	0.515	higher risk	3/3	No
11	TFAP2A	0.485	higher risk	3/3	No
12	MAGEA8	0.420	higher risk	3/3	No

Table S3. Selection frequencies with the univariable screen repeated inside each of 200 bootstrap replicates (fixed penalty 0.0597), shown for the 20 most frequently selected genes, alongside the frequencies from the locked selection.

Rank	Gene	In locked panel	Frequency, screen repeated	Frequency, locked run
1	FAIM2	Yes	0.730	0.785
2	NTSR1	Yes	0.680	0.725
3	KRT81	Yes	0.600	0.630
4	FLNC	Yes	0.585	0.600
5	CPS1	Yes	0.585	0.710
6	IGFBP1	Yes	0.580	0.625
7	CIDEC	Yes	0.445	0.560
8	TFAP2A	Yes	0.400	0.485
9	DSG3	No	0.390	
10	SLCO1B3	No	0.375	
11	CCL20	Yes	0.375	0.515
12	FBN2	Yes	0.370	0.540
13	MAGEA8	Yes	0.370	0.420
14	ABCC2	No	0.360	
15	TEX15	No	0.355	
16	UPK1B	No	0.345	
17	CTNND2	Yes	0.345	0.540
18	INSL4	No	0.310	
19	KRT14	No	0.295	
20	GRIK2	No	0.295	

Table S4. Univariable Cox coefficients, direction labels and TCGA-agreement flags for all 12 panel genes in TCGA-LUAD and each external cohort (48 gene-cohort rows).

Gene	Cohort	Cox coefficient	Direction	Matches TCGA
FAIM2	TCGA-LUAD	-0.2404	lower risk	Yes
FAIM2	GSE72094	0.0292	higher risk	No
FAIM2	GSE68465	-0.0833	lower risk	Yes
FAIM2	GSE31210	-0.1262	lower risk	Yes
NTSR1	TCGA-LUAD	0.1120	higher risk	Yes
NTSR1	GSE72094	0.1178	higher risk	Yes
NTSR1	GSE68465	0.1102	higher risk	Yes
NTSR1	GSE31210	-0.1918	lower risk	No
CPS1	TCGA-LUAD	0.0960	higher risk	Yes
CPS1	GSE72094	0.0737	higher risk	Yes
CPS1	GSE68465	0.0569	higher risk	Yes
CPS1	GSE31210	0.0226	higher risk	Yes
KRT81	TCGA-LUAD	0.1636	higher risk	Yes
KRT81	GSE72094	0.1073	higher risk	Yes
KRT81	GSE68465	0.0918	higher risk	Yes
KRT81	GSE31210	0.1163	higher risk	Yes
IGFBP1	TCGA-LUAD	0.1729	higher risk	Yes
IGFBP1	GSE72094	0.1914	higher risk	Yes

Gene	Cohort	Cox coefficient	Direction	Matches TCGA
IGFBP1	GSE68465	0.0556	higher risk	Yes
IGFBP1	GSE31210	0.1354	higher risk	Yes
FLNC	TCGA-LUAD	0.2683	higher risk	Yes
FLNC	GSE72094	0.2646	higher risk	Yes
FLNC	GSE68465	-0.0133	lower risk	No
FLNC	GSE31210	0.0114	higher risk	Yes
CIDEC	TCGA-LUAD	0.1111	higher risk	Yes
CIDEC	GSE72094	-0.0062	lower risk	No
CIDEC	GSE68465	-0.0495	lower risk	No
CIDEC	GSE31210	0.0213	higher risk	Yes
FBN2	TCGA-LUAD	0.1704	higher risk	Yes
FBN2	GSE72094	-0.0653	lower risk	No
FBN2	GSE68465	0.0176	higher risk	Yes
FBN2	GSE31210	0.3121	higher risk	Yes
CTNND2	TCGA-LUAD	-0.1618	lower risk	Yes
CTNND2	GSE72094	-0.1326	lower risk	Yes
CTNND2	GSE68465	-0.0651	lower risk	Yes
CTNND2	GSE31210	-0.1264	lower risk	Yes
CCL20	TCGA-LUAD	0.2154	higher risk	Yes
CCL20	GSE72094	0.0238	higher risk	Yes
CCL20	GSE68465	0.0780	higher risk	Yes
CCL20	GSE31210	0.4796	higher risk	Yes
TFAP2A	TCGA-LUAD	0.2887	higher risk	Yes
TFAP2A	GSE72094	0.2140	higher risk	Yes
TFAP2A	GSE68465	0.0706	higher risk	Yes
TFAP2A	GSE31210	0.4166	higher risk	Yes
MAGEA8	TCGA-LUAD	0.0802	higher risk	Yes
MAGEA8	GSE72094	0.0012	higher risk	Yes
MAGEA8	GSE68465	0.0063	higher risk	Yes
MAGEA8	GSE31210	0.1451	higher risk	Yes

Table S5. Univariable Cox hazard ratios per robust standard-deviation unit, with 95% confidence intervals, for every panel gene in every cohort. FDR is Benjamini-Hochberg corrected within each cohort.

Gene	Cohort	HR (95% CI)	p	FDR	Same direction as TCGA	CI excludes 1
FAIM2	TCGA-LUAD	0.79 (0.69–0.89)	<0.001	<0.001	Yes	Yes
FAIM2	GSE72094	1.03 (0.88–1.20)	0.708	0.944	No	No
FAIM2	GSE68465	0.92 (0.83–1.02)	0.124	0.342	Yes	No
FAIM2	GSE31210	0.88 (0.68–1.14)	0.336	0.551	Yes	No
NTSR1	TCGA-LUAD	1.12 (1.07–1.17)	<0.001	<0.001	Yes	Yes
NTSR1	GSE72094	1.13 (1.06–1.20)	<0.001	<0.001	Yes	Yes
NTSR1	GSE68465	1.12 (0.99–1.26)	0.064	0.256	Yes	No
NTSR1	GSE31210	0.82 (0.54–1.25)	0.368	0.551	No	No
CPS1	TCGA-LUAD	1.10 (1.04–1.17)	0.001	0.002	Yes	Yes
CPS1	GSE72094	1.08 (1.03–1.13)	0.003	0.008	Yes	Yes
CPS1	GSE68465	1.06 (1.00–1.12)	0.033	0.197	Yes	Yes
CPS1	GSE31210	1.02 (0.90–1.16)	0.727	0.873	Yes	No
KRT81	TCGA-LUAD	1.18 (1.09–1.27)	<0.001	<0.001	Yes	Yes
KRT81	GSE72094	1.11 (1.05–1.18)	<0.001	0.001	Yes	Yes
KRT81	GSE68465	1.10 (1.03–1.17)	0.007	0.085	Yes	Yes
KRT81	GSE31210	1.12 (0.92–1.38)	0.266	0.551	Yes	No
IGFBP1	TCGA-LUAD	1.19 (1.11–1.27)	<0.001	<0.001	Yes	Yes
IGFBP1	GSE72094	1.21 (1.11–1.33)	<0.001	<0.001	Yes	Yes
IGFBP1	GSE68465	1.06 (0.97–1.15)	0.199	0.353	Yes	No
IGFBP1	GSE31210	1.15 (0.87–1.51)	0.336	0.551	Yes	No
FLNC	TCGA-LUAD	1.31 (1.17–1.47)	<0.001	<0.001	Yes	Yes
FLNC	GSE72094	1.30 (1.09–1.56)	0.004	0.010	Yes	Yes
FLNC	GSE68465	0.99 (0.89–1.09)	0.806	0.880	No	No
FLNC	GSE31210	1.01 (0.76–1.34)	0.937	0.937	Yes	No
CIDEC	TCGA-LUAD	1.12 (1.07–1.17)	<0.001	<0.001	Yes	Yes
CIDEC	GSE72094	0.99 (0.84–1.17)	0.942	0.958	No	No
CIDEC	GSE68465	0.95 (0.84–1.08)	0.456	0.608	No	No
CIDEC	GSE31210	1.02 (0.86–1.21)	0.801	0.874	Yes	No
FBN2	TCGA-LUAD	1.19 (1.06–1.33)	0.004	0.005	Yes	Yes
FBN2	GSE72094	0.94 (0.80–1.09)	0.402	0.604	No	No
FBN2	GSE68465	1.02 (0.91–1.13)	0.750	0.880	Yes	No
FBN2	GSE31210	1.37 (1.10–1.70)	0.005	0.033	Yes	Yes
CTNND2	TCGA-LUAD	0.85 (0.76–0.95)	0.006	0.006	Yes	Yes
CTNND2	GSE72094	0.88 (0.78–0.99)	0.033	0.057	Yes	Yes
CTNND2	GSE68465	0.94 (0.86–1.02)	0.142	0.342	Yes	No
CTNND2	GSE31210	0.88 (0.68–1.14)	0.333	0.551	Yes	No
CCL20	TCGA-LUAD	1.24 (1.07–1.44)	0.005	0.006	Yes	Yes
CCL20	GSE72094	1.02 (0.84–1.25)	0.817	0.958	Yes	No
CCL20	GSE68465	1.08 (0.94–1.24)	0.279	0.419	Yes	No
CCL20	GSE31210	1.62 (1.24–2.10)	<0.001	0.004	Yes	Yes
TFAP2A	TCGA-LUAD	1.33 (1.17–1.52)	<0.001	<0.001	Yes	Yes
TFAP2A	GSE72094	1.24 (1.03–1.49)	0.022	0.044	Yes	Yes
TFAP2A	GSE68465	1.07 (0.96–1.20)	0.206	0.353	Yes	No
TFAP2A	GSE31210	1.52 (1.08–2.13)	0.015	0.061	Yes	Yes
MAGEA8	TCGA-LUAD	1.08 (1.03–1.14)	0.003	0.004	Yes	Yes
MAGEA8	GSE72094	1.00 (0.96–1.05)	0.958	0.958	Yes	No
MAGEA8	GSE68465	1.01 (0.91–1.11)	0.899	0.899	Yes	No
MAGEA8	GSE31210	1.16 (0.82–1.64)	0.413	0.551	Yes	No

Table S6. Descriptive internal cross-validation results, giving the per-fold C-index, test-set size and event count for every model. CPU models contributed three repeats of five folds; GPU models contributed one repeat of five folds.

Model	Repeat	Fold	n (test)	Events (test)	C-index
Clinical Cox	1	1	101	36	0.5176
Panel Cox	1	1	101	36	0.7329
Random survival forest	1	1	101	36	0.6734
Gradient boosting survival	1	1	101	36	0.6677
Clinical Cox	1	2	101	36	0.5508
Panel Cox	1	2	101	36	0.6467
Random survival forest	1	2	101	36	0.6537
Gradient boosting survival	1	2	101	36	0.6164
Clinical Cox	1	3	101	37	0.5336
Panel Cox	1	3	101	37	0.6009
Random survival forest	1	3	101	37	0.6193
Gradient boosting survival	1	3	101	37	0.6052
Clinical Cox	1	4	101	37	0.4597
Panel Cox	1	4	101	37	0.6868
Random survival forest	1	4	101	37	0.6947
Gradient boosting survival	1	4	101	37	0.6847
Clinical Cox	1	5	100	36	0.5564
Panel Cox	1	5	100	36	0.7141
Random survival forest	1	5	100	36	0.6979
Gradient boosting survival	1	5	100	36	0.7053
Clinical Cox	2	1	101	36	0.4511
Panel Cox	2	1	101	36	0.6428
Random survival forest	2	1	101	36	0.6387
Gradient boosting survival	2	1	101	36	0.6567
Clinical Cox	2	2	101	36	0.5470
Panel Cox	2	2	101	36	0.6209
Random survival forest	2	2	101	36	0.6846
Gradient boosting survival	2	2	101	36	0.6551
Clinical Cox	2	3	101	37	0.4101
Panel Cox	2	3	101	37	0.6312
Random survival forest	2	3	101	37	0.6295
Gradient boosting survival	2	3	101	37	0.6240
Clinical Cox	2	4	101	37	0.4532
Panel Cox	2	4	101	37	0.7169
Random survival forest	2	4	101	37	0.7309
Gradient boosting survival	2	4	101	37	0.6906

Model	Repeat	Fold	n (test)	Events (test)	C-index
Clinical Cox	2	5	100	36	0.5465
Panel Cox	2	5	100	36	0.7561
Random survival forest	2	5	100	36	0.6956
Gradient boosting survival	2	5	100	36	0.7106
Clinical Cox	3	1	101	36	0.5113
Panel Cox	3	1	101	36	0.6967
Random survival forest	3	1	101	36	0.6854
Gradient boosting survival	3	1	101	36	0.6894
Clinical Cox	3	2	101	36	0.4126
Panel Cox	3	2	101	36	0.6614
Random survival forest	3	2	101	36	0.6703
Gradient boosting survival	3	2	101	36	0.6429
Clinical Cox	3	3	101	37	0.5240
Panel Cox	3	3	101	37	0.6470
Random survival forest	3	3	101	37	0.6604
Gradient boosting survival	3	3	101	37	0.6244
Clinical Cox	3	4	101	37	0.5493
Panel Cox	3	4	101	37	0.7478
Random survival forest	3	4	101	37	0.7532
Gradient boosting survival	3	4	101	37	0.7261
Clinical Cox	3	5	100	36	0.4937
Panel Cox	3	5	100	36	0.6239
Random survival forest	3	5	100	36	0.6064
Gradient boosting survival	3	5	100	36	0.6407
DeepSurv	1	1	101	36	0.6313
SurvivalPFN	1	1	101	36	0.6463
DeepSurv	1	2	101	36	0.6041
SurvivalPFN	1	2	101	36	0.6839
DeepSurv	1	3	101	37	0.7097
SurvivalPFN	1	3	101	37	0.7250
DeepSurv	1	4	101	37	0.6105
SurvivalPFN	1	4	101	37	0.7049
DeepSurv	1	5	100	36	0.6519
SurvivalPFN	1	5	100	36	0.6481

Table S7. Overall survival by tertile of the panel Cox score, with cut-points fixed in TCGA. Survival is the Kaplan-Meier estimate in percent with 95% confidence interval; the last row for each cohort gives the Cox hazard ratio for the high- versus low-risk group.

Cohort	Risk group	n	Deaths	3-year OS, %	5-year OS, %
GSE72094	Low	115	23	69.9 (54.9–80.7)	43.5 (16.9–67.7)
GSE72094	Intermediate	155	42	67.4 (57.6–75.4)	65.1 (54.6–73.8)
GSE72094	High	128	48	51.3 (38.4–62.8)	34.2 (13.8–55.9)
GSE72094	High vs low, HR (95% CI)	398	113	2.39 (1.45–3.94)	p = 0.0006
GSE68465	Low	156	70	77.1 (69.6–83.0)	62.6 (53.9–70.2)
GSE68465	Intermediate	175	103	63.8 (56.0–70.5)	50.0 (42.0–57.5)
GSE68465	High	111	63	60.3 (50.6–68.7)	51.5 (41.5–60.6)
GSE68465	High vs low, HR (95% CI)	442	236	1.43 (1.02–2.01)	p = 0.0397
GSE31210	Low	97	9	94.8 (87.8–97.8)	89.9 (81.4–94.6)
GSE31210	Intermediate	60	6	94.6 (84.2–98.2)	88.1 (75.3–94.5)
GSE31210	High	69	20	82.1 (70.6–89.4)	71.9 (58.3–81.7)
GSE31210	High vs low, HR (95% CI)	226	35	3.65 (1.66–8.03)	p = 0.0013

Table S8. Paired bootstrap comparisons of SurvivalPFN against the age and sex Cox model and against panel Cox in each cohort, with the delta C-index, its 95% confidence interval and the number of paired replicates.

Cohort	Model	Comparator	ΔC	CI lower	CI upper	Replicates
GSE31210	SurvivalPFN	Clinical Cox	0.0675	-0.0377	0.1794	2000
GSE31210	SurvivalPFN	Panel Cox	0.0056	-0.0259	0.0394	2000
GSE68465	SurvivalPFN	Clinical Cox	0.0049	-0.0531	0.0621	2000
GSE68465	SurvivalPFN	Panel Cox	0.0055	-0.0088	0.0204	2000
GSE72094	SurvivalPFN	Clinical Cox	0.0864	0.0094	0.1661	2000
GSE72094	SurvivalPFN	Panel Cox	0.0106	-0.0142	0.0355	2000

Table S9. Association of the standardized panel Cox and SurvivalPFN scores with survival within each external cohort, adjusted for categorical stage, and for stage, age and sex. HR is per standard deviation; p values are from likelihood-ratio tests of the added score. The last rows give panel Cox discrimination among stage I patients.

Cohort	Score	n/events	HR, stage-adjusted	p	HR, stage, age and sex adjusted	p
GSE72094	Panel Cox score	393/111	1.36 (1.12–1.65)	0.002	1.38 (1.13–1.68)	0.002
GSE72094	SurvivalPFN score	393/111	1.41 (1.15–1.73)	<0.001	1.38 (1.13–1.69)	0.001
GSE68465	Panel Cox score	439/235	1.12 (0.98–1.28)	0.087	1.11 (0.97–1.27)	0.121
GSE68465	SurvivalPFN score	439/235	1.19 (1.04–1.36)	0.010	1.20 (1.05–1.36)	0.008
GSE31210	Panel Cox score	226/35	1.56 (1.11–2.17)	0.009	1.51 (1.07–2.14)	0.019
GSE31210	SurvivalPFN score	226/35	1.56 (1.09–2.22)	0.011	1.52 (1.05–2.18)	0.023
GSE72094	Panel Cox C-index within stage I	254/52	0.607 (0.525–0.688)			
GSE68465	Panel Cox C-index within stage I	276/111	0.556 (0.498–0.615)			
GSE31210	Panel Cox C-index within stage I	168/17	0.703 (0.566–0.822)			

Table S10. Calibration of transported models. IBS is the integrated Brier score with a 1,000-replicate bootstrap interval where shown; the reference is a covariate-free Kaplan-Meier estimate in the target cohort; IPA is the index of prediction accuracy (1 − IBS/reference IBS); the calibration slope is the coefficient of the transported prognostic index (3-year complementary log-log risk for SurvivalPFN) in the target cohort; O/E is observed over mean predicted 3-year risk.

Cohort	Model	IBS (95% CI)	KM reference IBS	IPA, %	Calibration slope (95% CI)	O/E 3-year
GSE72094	Panel Cox	0.1435 (0.1261–0.1602)	0.1491	3.8	0.56 (0.29–0.84)	1.02
GSE72094	SurvivalPFN (raw)	0.1445 (0.1256–0.1627)	0.1491	3.1	0.51 (0.27–0.76)	1.10
GSE72094	SurvivalPFN (conformalized)	0.1515 (0.1349–0.1680)	0.1491	-1.6	0.57 (0.28–0.86)	0.98
GSE72094	Stage-only Cox (staged patients)	0.1390	0.1476	5.9	0.94 (0.60–1.29)	1.05
GSE72094	Stage + panel Cox (staged patients)	0.1347	0.1476	8.8	0.70 (0.48–0.93)	1.06
GSE68465	Panel Cox	0.1997 (0.1854–0.2148)	0.1929	-3.5	0.28 (0.08–0.47)	1.00
GSE68465	SurvivalPFN (raw)	0.1977 (0.1808–0.2155)	0.1929	-2.4	0.29 (0.12–0.47)	1.08
GSE68465	SurvivalPFN (conformalized)	0.1961 (0.1817–0.2115)	0.1929	-1.6	0.34 (0.15–0.54)	0.94
GSE68465	Stage-only Cox (staged patients)	0.1708	0.1929	11.5	1.48 (1.18–1.79)	0.95

Cohort	Model	IBS (95% CI)	KM reference IBS	IPA, %	Calibration slope (95% CI)	O/E 3-year
GSE68465	Stage + panel Cox (staged patients)	0.1777	0.1929	7.9	0.67 (0.47–0.87)	1.05
GSE31210	Panel Cox	0.1797 (0.1563–0.2046)	0.0938	-91.6	0.78 (0.38–1.17)	0.27
GSE31210	SurvivalPFN (raw)	0.1424 (0.1224–0.1634)	0.0938	-51.9	0.79 (0.36–1.22)	0.33
GSE31210	SurvivalPFN (conformalized)	0.1573 (0.1391–0.1769)	0.0938	-67.7	0.92 (0.42–1.43)	0.28
GSE31210	Stage-only Cox (staged patients)	0.1409	0.0938	-50.3	2.80 (1.51–4.09)	0.31
GSE31210	Stage + panel Cox (staged patients)	0.1425	0.0938	-52.0	1.00 (0.57–1.42)	0.32

Table S11. Calibration metrics for raw and conformalized SurvivalPFN predictions in each cohort. The 10-bin D-calibration histogram behind each chi-squared statistic is plotted in full as Figure S7 and retained in the accompanying CSV.

Cohort	Model	n	Events	C-index	IBS	Brier 1 y	Brier 3 y	D-cal χ^2	D-cal p
GSE72094	SurvivalPFN-split	398	113	0.6287	0.14450	0.0975	0.2217	3.103	0.9601
GSE72094	CSD-SurvivalPFN	398	113	0.6289	0.15153	0.1086	0.2212	11.913	0.2183
GSE68465	SurvivalPFN-split	442	236	0.5867	0.19767	0.0994	0.2266	20.393	0.01564
GSE68465	CSD-SurvivalPFN	442	236	0.5866	0.19611	0.1047	0.2233	7.784	0.5561
GSE31210	SurvivalPFN-split	226	35	0.6706	0.14245	0.0623	0.1298	56.935	5.20e-09
GSE31210	CSD-SurvivalPFN	226	35	0.6709	0.15730	0.0872	0.1467	79.330	2.19e-13

Table S12. Extended discrimination metrics for every model and cohort, giving Uno's C-index, the truncation time in days and cumulative/dynamic area under the curve at 1, 3 and 5 years. Empty cells were not estimable given the available censoring and event support.

Cohort	Model	Uno C	τ (days)	AUC 1 y	AUC 3 y	AUC 5 y
GSE31210	Clinical Cox	0.5697	1667.0	0.6436	0.6836	NE
GSE31210	DeepSurv	0.6094	1667.0	0.6824	0.6304	NE
GSE31210	Gradient boosting survival	0.6513	1667.0	0.6678	0.6510	NE
GSE31210	Panel Cox	0.6431	1667.0	0.5935	0.6838	NE
GSE31210	Random survival forest	0.6067	1667.0	0.6002	0.6071	NE
GSE31210	SurvivalPFN	0.6531	1667.0	0.6014	0.6728	NE
GSE68465	Clinical Cox	0.5748	2381.7	0.6022	0.5959	0.5927
GSE68465	DeepSurv	0.5318	2381.7	0.5909	0.5579	0.5240
GSE68465	Gradient boosting survival	0.5601	2381.7	0.6344	0.5755	0.5505
GSE68465	Panel Cox	0.5726	2381.7	0.6614	0.5979	0.5633
GSE68465	Random survival forest	0.5660	2381.7	0.6387	0.5929	0.5627
GSE68465	SurvivalPFN	0.5795	2381.7	0.6609	0.6077	0.5789
GSE72094	Clinical Cox	0.5426	1035.8	0.5545	NE	NE
GSE72094	DeepSurv	0.5458	1035.8	0.5995	NE	NE
GSE72094	Gradient boosting survival	0.5871	1035.8	0.6154	NE	NE
GSE72094	Panel Cox	0.5980	1035.8	0.6281	NE	NE
GSE72094	Random survival forest	0.5973	1035.8	0.6086	NE	NE
GSE72094	SurvivalPFN	0.6092	1035.8	0.6392	NE	NE

Table S13. Paired tumor-normal results for all 12 genes in the 58 matched pairs, giving the median tumor and normal values on the variance-stabilized scale, the median paired difference, paired Cohen's dz, the Wilcoxon signed-rank statistic, the raw p value and the Benjamini-Hochberg false discovery rate. Direction is shown only for genes with a false discovery rate below 0.05; CIDEC did not differ and is shown as unchanged.

Gene	Pairs	Median tumor	Median normal	Median difference	Cohen dz	Wilcoxon W	p	FDR	Direction in tumor
FAIM2	58	6.650	7.405	-0.621	-0.23	516	0.008576	0.01286	Down
NTSR1	58	4.631	4.481	0.169	0.41	436	0.003148	0.007069	Up
CPS1	58	7.101	7.005	0.335	0.45	485	0.004124	0.007069	Up
KRT81	58	6.338	4.982	1.174	0.84	73	1.37e-09	8.25e-09	Up
IGFBP1	58	4.877	4.585	0.199	0.37	547	0.02637	0.02916	Up
FLNC	58	9.550	10.165	-0.507	-0.27	550	0.01802	0.02402	Down
CIDEC	58	4.378	4.366	0.000	0.14	765	0.9666	0.9666	No change (FDR 0.97)
FBN2	58	6.975	6.153	0.592	0.56	325	4.00e-05	1.20e-04	Up
CTNND2	58	5.313	8.321	-2.837	-0.97	203	4.37e-07	1.75e-06	Down
CCL20	58	8.965	7.866	0.834	0.41	479	0.003557	0.007069	Up
TFAP2A	58	8.805	5.756	2.790	1.60	13	6.89e-11	8.27e-10	Up
MAGEA8	58	4.257	4.248	0.025	0.30	408	0.02673	0.02916	Up

Table S14. Stage trend results for all 12 genes across the 496 patients with TCGA-CDR pathological stage, giving Spearman's rho, the raw p value and the false discovery rate.

Gene	Patients	Spearman rho	p	FDR
FAIM2	496	−0.1691	0.000155	0.000928
NTSR1	496	0.0514	0.253	0.276
CPS1	496	0.0919	0.0408	0.0815
KRT81	496	0.0656	0.144	0.173
IGFBP1	496	0.1820	4.56e-05	0.000547
FLNC	496	0.1385	0.00199	0.00597
CIDEC	496	0.1529	0.000634	0.00253
FBN2	496	0.0768	0.0874	0.116
CTNND2	496	−0.1182	0.00841	0.0202
CCL20	496	0.0830	0.0646	0.0969
TFAP2A	496	0.0848	0.059	0.0969
MAGEA8	496	−0.0062	0.89	0.89

Table S15. Functional enrichment output from g:Profiler across Gene Ontology, Reactome, KEGG, WikiPathways and CORUM. The query returned an empty result set, which is reported here rather than omitted. This query used the whole genome as background; the analysis against the 1,232-gene selection universe is given in Table S17.

Result
No Gene Ontology, Reactome, KEGG, WikiPathways or CORUM term reached significance for the 12-gene query.

Table S16. STRING v12 network output at medium confidence 0.4. The edge list returned empty, which is reported here rather than omitted.

Result
No protein-protein interaction was returned among the 12 panel proteins at medium confidence 0.4 (PPI enrichment p = 1.0).

Table S17. Functional enrichment of the 12 panel genes against the 1,232-gene selection universe as background. For each source the number of terms tested, the number with adjusted p < 0.05, the smallest adjusted p value and the top-ranked term are shown.

Method	Source	Terms tested	Adjusted p < 0.05	Smallest adjusted p	Top term
g:Profiler, g:SCS	CORUM	3	0	1	CORUM root (140 genes in universe)
g:Profiler, g:SCS	GO:BP	652	0	1	urea cycle (1 gene in universe)
g:Profiler, g:SCS	GO:CC	107	0	1	Golgi membrane (36 genes in universe)
g:Profiler, g:SCS	GO:MF	116	0	1	nucleotide binding (104 genes in universe)
g:Profiler, g:SCS	KEGG	20	0	1	KEGG root term (722 genes in universe)
g:Profiler, g:SCS	REAC	55	0	1	ATF4 activates genes in response to endoplasmic reticulum stress (1 gene in universe)
g:Profiler, g:SCS	WP	40	0	1	WIKIPATHWAYS (823 genes in universe)
g:Profiler, Benjamini-Hochberg	CORUM	3	2	0.0147	RB1-TFAP2A complex (1 gene in universe)
g:Profiler, Benjamini-Hochberg	GO:BP	652	0	0.172	negative regulation of release of sequestered calcium ion into cytosol (1 gene in universe)
g:Profiler, Benjamini-Hochberg	GO:CC	107	0	0.349	mitochondrial nucleoid (1 gene in universe)
g:Profiler, Benjamini-Hochberg	GO:MF	116	0	0.227	potassium ion binding (1 gene in universe)
g:Profiler, Benjamini-Hochberg	KEGG	20	0	0.212	Biosynthesis of amino acids (8 genes in universe)
g:Profiler, Benjamini-Hochberg	REAC	55	0	0.0672	Cell-extracellular matrix interactions (1 gene in universe)
g:Profiler, Benjamini-Hochberg	WP	40	0	0.0652	Pyrimidine metabolism and related diseases (1 gene in universe)
MSigDB hypergeometric, BH	c2.cp.kegg_medicus	24	0	1	KEGG_MEDICUS_REFERENCE_GPCR_PLCB_ITPR_SIGNALING_PATHWAY (NTSR1)
MSigDB hypergeometric, BH	c2.cp.reactome	340	0	1	REACTOME_CELL_JUNCTION_ORGANIZATION (FLNC;TFAP2A)
MSigDB hypergeometric, BH	h.all	38	0	1	HALLMARK_APICAL_JUNCTION (FLNC)

Table S18. Outcome of the five substantive prespecified interpretation criteria and the procedural sixth step. Three of the five substantive criteria were met.

Criterion	Result

Criterion	Result
SurvivalPFN point estimate exceeds age/sex Cox in both primary cohorts	Pass
Paired bootstrap delta-C CI excludes zero in at least one primary cohort	Pass
CSD preserves or improves IBS in both primary cohorts	Fail
Primary-cohort gene-direction concordance is at least 75%	Pass
Every panel gene has selection frequency at least 0.60	Fail
Frozen model is scored in the early-stage cohort	Completed (procedural; not counted)

Table S19. External discrimination for the three post-lock sensitivity models in each cohort, with 95% bootstrap confidence intervals. Every row in this table was specified after the primary model lock.

Cohort	Model	n	Events	C-index	CI lower	CI upper
GSE72094	XGBoost Cox	398	113	0.5661	0.5096	0.6302
GSE72094	SurvivalPFN + age/sex	398	113	0.6352	0.5787	0.6951
GSE72094	Stage-only Cox	398	113	0.6296	0.5792	0.6785
GSE68465	XGBoost Cox	442	236	0.5558	0.5120	0.5958
GSE68465	SurvivalPFN + age/sex	442	236	0.5948	0.5545	0.6333
GSE31210	XGBoost Cox	226	35	0.6336	0.5473	0.7249
GSE31210	SurvivalPFN + age/sex	226	35	0.6672	0.5818	0.7544
GSE31210	Stage-only Cox	226	35	0.6776	0.5912	0.7596

Table S20. Sensitivity analyses specified after the lock. C-index values are Harrell's C with 95% bootstrap intervals; differences are paired against the corresponding locked 12-gene model. The last rows summarize the GSE72094/GSE68465 overlap checks, in which sites other than Moffitt cannot overlap with GSE72094 and act as the reference.

Analysis	Cohort	Model	C-index (95% CI) or result	Difference vs locked (95% CI)
Six stable genes	GSE72094	Panel Cox	0.621 (0.562–0.674)	0.001 (−0.036 to 0.039)
Six stable genes	GSE72094	Random survival forest	0.612 (0.553–0.670)	
Six stable genes	GSE72094	Gradient boosting survival	0.589 (0.531–0.644)	
Six stable genes	GSE68465	Panel Cox	0.572 (0.529–0.616)	−0.005 (−0.038 to 0.028)
Six stable genes	GSE68465	Random survival forest	0.554 (0.514–0.594)	
Six stable genes	GSE68465	Gradient boosting survival	0.554 (0.514–0.594)	
Six stable genes	GSE31210	Panel Cox	0.555 (0.451–0.664)	−0.104 (−0.179 to −0.036)
Six stable genes	GSE31210	Random survival forest	0.440 (0.339–0.548)	
Six stable genes	GSE31210	Gradient boosting survival	0.462 (0.365–0.562)	
Six stable genes	GSE72094	SurvivalPFN	0.630 (0.572–0.688)	−0.001 (−0.038 to 0.037)
Six stable genes	GSE68465	SurvivalPFN	0.583 (0.540–0.624)	−0.001 (−0.034 to 0.032)
Six stable genes	GSE31210	SurvivalPFN	0.550 (0.441–0.660)	−0.115 (−0.184 to −0.051)
Expression filter, bottom 10% removed (8 genes kept)	GSE72094	Panel Cox	0.607 (0.547–0.660)	−0.013 (−0.031 to 0.004)
Expression filter, bottom 10% removed (8 genes kept)	GSE68465	Panel Cox	0.566 (0.524–0.605)	−0.012 (−0.022 to −0.001)
Expression filter, bottom 10% removed (8 genes kept)	GSE31210	Panel Cox	0.650 (0.556–0.740)	−0.010 (−0.025 to 0.004)
Expression filter, bottom 25% removed (3 genes kept)	GSE72094	Panel Cox	0.588 (0.530–0.641)	−0.032 (−0.083 to 0.022)
Expression filter, bottom 25% removed (3 genes kept)	GSE68465	Panel Cox	0.519 (0.474–0.559)	−0.059 (−0.096 to −0.023)
Expression filter, bottom 25% removed (3 genes kept)	GSE31210	Panel Cox	0.590 (0.486–0.690)	−0.070 (−0.160 to 0.019)
DeepSurv, validation-based early stopping	GSE72094	DeepSurv	0.638 (0.581–0.692)	locked rule: 0.569
DeepSurv, validation-based early stopping	GSE68465	DeepSurv	0.591 (0.550–0.630)	locked rule: 0.540
DeepSurv, validation-based early stopping	GSE31210	DeepSurv	0.643 (0.544–0.745)	locked rule: 0.624
Excluding 17 flattened non-adenocarcinoma labels	GSE72094	Panel Cox	0.619	locked: 0.620
Excluding 17 flattened non-adenocarcinoma labels	GSE68465	Panel Cox	0.579	locked: 0.578
Excluding 17 flattened non-adenocarcinoma labels	GSE31210	Panel Cox	0.656	locked: 0.659
TCGA-CDR overall survival	GSE72094	Panel Cox	0.620	locked: 0.620

Analysis	Cohort	Model	C-index (95% CI) or result	Difference vs locked (95% CI)
TCGA-CDR overall survival	GSE68465	Panel Cox	0.578	locked: 0.578
TCGA-CDR overall survival	GSE31210	Panel Cox	0.659	locked: 0.659
GSE68465 without Moffitt (HLM) patients	n = 363; 176 deaths	SurvivalPFN	0.595 (0.551–0.639)	
GSE68465 without Moffitt (HLM) patients	n = 363; 176 deaths	Panel Cox	0.588 (0.543–0.633)	
GSE68465 without Moffitt (HLM) patients	n = 363; 176 deaths	Age/sex Cox	0.581 (0.531–0.630)	
Overlap check: clinically identical records	GSE68465 vs GSE72094		Moffitt 3/79; other sites 26/363	
Overlap check: standardized best-match similarity	GSE68465 vs GSE72094		Moffitt max 5.29; other sites max 5.47	median r 0.33 vs 0.28

Table S21. External C-index of the locked panel against 1,000 random 12-gene panels drawn from the 1,232-gene selection universe, each fitted with the panel Cox specification in TCGA. The empirical p value is the proportion of random panels with a C-index at least as high as the locked panel.

Cohort	Locked panel C	Random median (IQR)	Random 2.5th–97.5th	Percentile of locked	Empirical p	Random > 0.60
GSE72094	0.620	0.601 (0.580–0.622)	0.536–0.654	73	0.27	51%
GSE68465	0.578	0.566 (0.547–0.582)	0.513–0.613	70	0.30	10%
GSE31210	0.659	0.598 (0.551–0.633)	0.464–0.695	90	0.11	49%

Table S22. Proportional hazards tests based on scaled Schoenfeld residuals against rank-transformed time. The global statistic for the 12-gene TCGA model is the sum of the 12 one-degree-of-freedom statistics.

Cohort	Model	Term	Test statistic	p
TCGA-LUAD	12-gene multivariable Cox	FAIM2	0.086	0.770
TCGA-LUAD	12-gene multivariable Cox	NTSR1	1.299	0.254
TCGA-LUAD	12-gene multivariable Cox	CPS1	3.059	0.080
TCGA-LUAD	12-gene multivariable Cox	KRT81	0.017	0.895
TCGA-LUAD	12-gene multivariable Cox	IGFBP1	0.001	0.972
TCGA-LUAD	12-gene multivariable Cox	FLNC	1.306	0.253
TCGA-LUAD	12-gene multivariable Cox	CIDEC	2.614	0.106
TCGA-LUAD	12-gene multivariable Cox	FBN2	0.002	0.965
TCGA-LUAD	12-gene multivariable Cox	CTNND2	0.298	0.585
TCGA-LUAD	12-gene multivariable	CCL20	0.271	0.603

Cohort	Model	Term	Test statistic	p
	Cox			
TCGA-LUAD	12-gene multivariable Cox	TFAP2A	1.741	0.187
TCGA-LUAD	12-gene multivariable Cox	MAGEA8	1.621	0.203
TCGA-LUAD	12-gene multivariable Cox	GLOBAL (sum of 12 1-df statistics)	12.317	0.421
TCGA-LUAD	Locked panel Cox score	panel_score	1.428	0.232
TCGA-LUAD	Stage + panel score	stage	0.467	0.494
TCGA-LUAD	Stage + panel score	panel_score	1.302	0.254
GSE72094	Locked panel Cox score	panel_score	3.675	0.055
GSE72094	Stage + panel score	stage	0.033	0.856
GSE72094	Stage + panel score	panel_score	3.231	0.072
GSE68465	Locked panel Cox score	panel_score	8.273	0.004
GSE68465	Stage + panel score	stage	2.275	0.132
GSE68465	Stage + panel score	panel_score	8.832	0.003
GSE31210	Locked panel Cox score	panel_score	0.296	0.587
GSE31210	Stage + panel score	stage	8.637	0.003
GSE31210	Stage + panel score	panel_score	0.214	0.644
